# Aptamers targeting SARS-CoV-2 nucleocapsid protein exhibit potential anti pan-coronavirus activity

**DOI:** 10.1038/s41392-024-01748-w

**Published:** 2024-02-14

**Authors:** Minghui Yang, Chunhui Li, Guoguo Ye, Chenguang Shen, Huiping Shi, Liping Zhong, Yuxin Tian, Mengyuan Zhao, Pengfei Wu, Abid Hussain, Tian Zhang, Haiyin Yang, Jun Yang, Yuhua Weng, Xinyue Liu, Zhimin Wang, Lu Gan, Qianyu Zhang, Yingxia Liu, Ge Yang, Yuanyu Huang, Yongxiang Zhao

**Affiliations:** 1 School of Life Science, Advanced Research Institute of Multidisciplinary Science, Key Laboratory of Molecular Medicine and Biotherapy, Beijing Institute of Technology, Beijing, 100081 China; 2 National Clinical Research Center for infectious disease, Shenzhen Third People’s Hospital, Second Hospital Affiliated to Southern University of Science and Technology, Shenzhen, 518112 China; 3 Guangdong Provincial Key Laboratory of Tropical Disease Research, School of Public Health, Southern Medical University, Guangzhou, 510515 China; 4 State Key Laboratory of Targeting Oncology, National Center for International Research of Biotargeting Theranostics, Guangxi Key Laboratory of Biotargeting Theranostics, Collaborative Innovation Center for Targeting Tumor Diagnosis and Therapy, Guangxi Medical University, Nanning, 530021 China; 5 CAMS Key Laboratory of Antiviral Drug Research, Beijing Key Laboratory of Antimicrobial Agents, Institute of Medicinal Biotechnology, Chinese Academy of Medical Sciences and Peking Union Medical College, Beijing, 100730 China

**Keywords:** Drug development, Target validation

## Abstract

Emerging and recurrent infectious diseases caused by human coronaviruses (HCoVs) continue to pose a significant threat to global public health security. In light of this ongoing threat, the development of a broad-spectrum drug to combat HCoVs is an urgently priority. Herein, we report a series of anti-pan-coronavirus ssDNA aptamers screened using Systematic Evolution of Ligands by Exponential Enrichment (SELEX). These aptamers have nanomolar affinity with the nucleocapsid protein (NP) of Severe acute respiratory syndrome coronavirus 2 (SARS-CoV-2) and also show excellent binding efficiency to the N proteins of both SARS, MERS, HCoV-OC43 and -NL63 with affinity K_D_ values of 1.31 to 135.36 nM. Such aptamer-based therapeutics exhibited potent antiviral activity against both the authentic SARS-CoV-2 prototype strain and the Omicron variant (BA.5) with EC_50_ values at 2.00 nM and 41.08 nM, respectively. The protein docking analysis also evidenced that these aptamers exhibit strong affinities for N proteins of pan-coronavirus and other HCoVs (−229E and -HKU1). In conclusion, we have identified six aptamers with a high pan-coronavirus antiviral activity, which could potentially serve as an effective strategy for preventing infections by unknown coronaviruses and addressing the ongoing global health threat.

## Introduction

The Coronavirus (CoV) stands out as the largest single-stranded RNA virus within its genome. Among the seven coronaviruses known to infect humans, Severe acute respiratory syndrome coronavirus (SARS), Middle east respiratory syndrome coronavirus (MERS) and SARS-CoV-2 caused global outbreaks in 2002, 2012 and 2019–2022,^1–3^ respectively. These three prominent HCoVs^4^ share similar symptoms, such as fever, shortness of breath or dyspnea, muscle pain, pneumonia, and even a high mortality rate in severe cases.^5^ In addition, HCoV-OC43, -NL63, −229E and -HKU1 are also classified as HCoVs, which have received less attention for causing self-healing disease.^6^ The COVID-19 crisis has been considered a “gray rhino” rather than a “black swan” as it represented a highly probable but neglected threat with enormous impact, highlighting the likelihood novel unknown Coronaviruses continuing to emerge as significant human pathogens.^7^ The Corona Virus Disease 2019 (COVID-19) pandemic has demonstrated that the traditional antiviral strategy of “One virus, One drug” is ineffective in controlling rapidly disseminating outbreaks. This emphasizes the urgent need to identify broad-spectrum agents with pan-coronaviral activities.

Developing effective antiviral drugs involves strategies focused on blocking viral entry into host cells and inhibiting viral replication/transcription. A commonly employed approach is targeting the receptor-binding domain of viruses to block viral invasion. For instance, neutralizing antibodies like Sotrovimab,^8^ Bebtelovimab,^9^ Tixagevimab & Cilgavimab,^10^ Bamlanivimab & Etesevimab,^11^ and Casirivimab & Imdevimab^12^ have been developed for SARS-CoV-2 by targeting its Spike protein (SP) to inhibit virus entry into host cells. Although these antibodies are highly virus-neutralizing, the large number of mutations in the S protein recognition epitope can reduce or even negate their effectiveness.^13–15^ In addition to these structural proteins, SARS-CoV-2 encodes 16 nonstructural proteins (nsp1-16) and 9 accessory proteins responsible for viral replication and transcription. Small molecule inhibitors such as Remdesivir, developed for the Ebola virus, and Molnupiravir, developed for the Venezuelan equine encephalitis virus, have been proposed for SARS-CoV-2 inhibition by targeting RNA-dependent RNA polymerase (RdRp).^16,17^ Additionally, nirmatrelvir in Paxlovid was also designed for SARS-CoV-2 by targeting Mpro.^18,19^ Despite several therapeutic agents currently being evaluated, none seems to provide a clear path to a cure. The Nuclecapsid protein, a component of the ribonucleoprotein core encoded by coronaviruses,^20^ plays a crucial role in virus invasion, assembly, and extra-cellular processes.^20–22^ Among all CoV-encoded proteins, the N protein demonstrates the highest degree of conservation, particularly in its C-terminal domain (CTD) and N-terminal domain (NTD).^23^ Furthermore, the N proteins of SARS-CoV-2, SARS, and MERS exhibit not only sequence conservation but also conservation in secondary structure.^24–26^ Given its evolutionary conservation and pivotal role in viral replication, the N protein emerges as a promising target for the discovery of antiviral drugs.

Nucleic acid modalities, such as aptamer oligonucleotides, present a promising avenue as advanced solutions for disease diagnosis and treatment. Aptamers, often referred to as “chemical antibodies”, are innovative biomolecules known for their heightened specificity and affinity, making them valuable in the realm of antiviral research.^27–29^ Regarding the target of the N protein in SARS-CoV-2, there have been some reports of N protein aptamers being used for virus detection.^30,31^ However, there is still a lack of N protein-targeting aptamers for antiviral therapy.

In this study, we are presenting six potent “pan-coronavirus” antiviral aptamers that function by blocking the N protein of HCoVs. The affinity with the N protein of SARS-CoV-2, SARS, MERS, HCoV-OC43, -NL63 other HCoVs, antiviral activity against authentic SARS-CoV-2 and its variants as well as -OC43 were broadly investigated and discussed. This approach presents a novel way to develop broad-spectrum anti-coronavirus drugs and have a strategic potential against unknown coronavirus.

## Results

### Enrichment and identification of aptamers targeting SARS-CoV-2 N protein

**Fig. 1:**
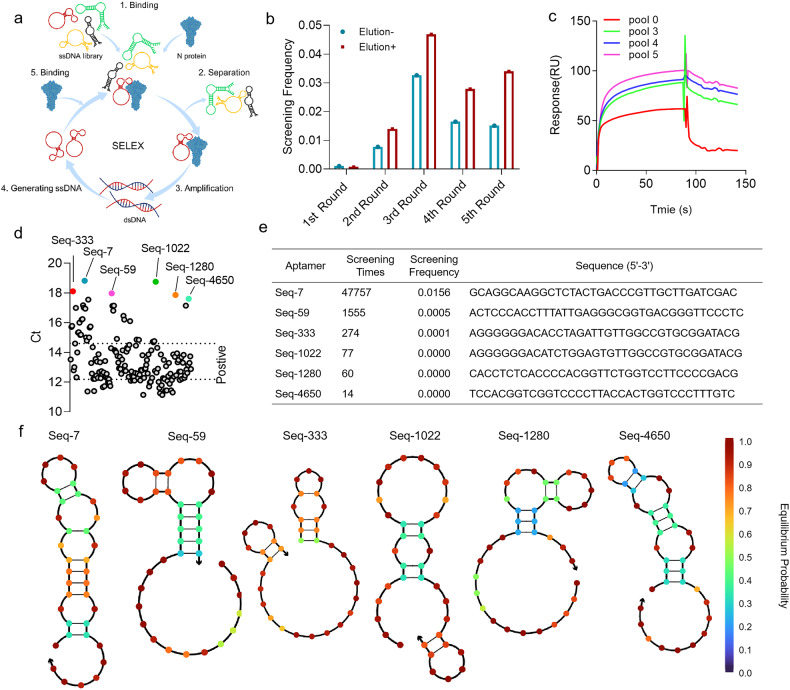
Identification of aptamers of SARS-CoV-2 N protein with high neutralization activity. **a** Schematic diagram of magnetic bead screening aptamers. **b** The retention rate of five rounds of screening aptamers using SARS-CoV-2 NP. **c** Detection of library affinity using SPR (Surface plasmon resonance). **d** Preliminary screening of the antiviral effects of 165 aptamers against authentic SARS-CoV-2, 6 of 165 aptamers were selected for further evaluation. **e** The screening details and sequences of 6 selected aptamers. **f** The secondary structures of six aptamers using NUPACK software

**Fig. 2:**
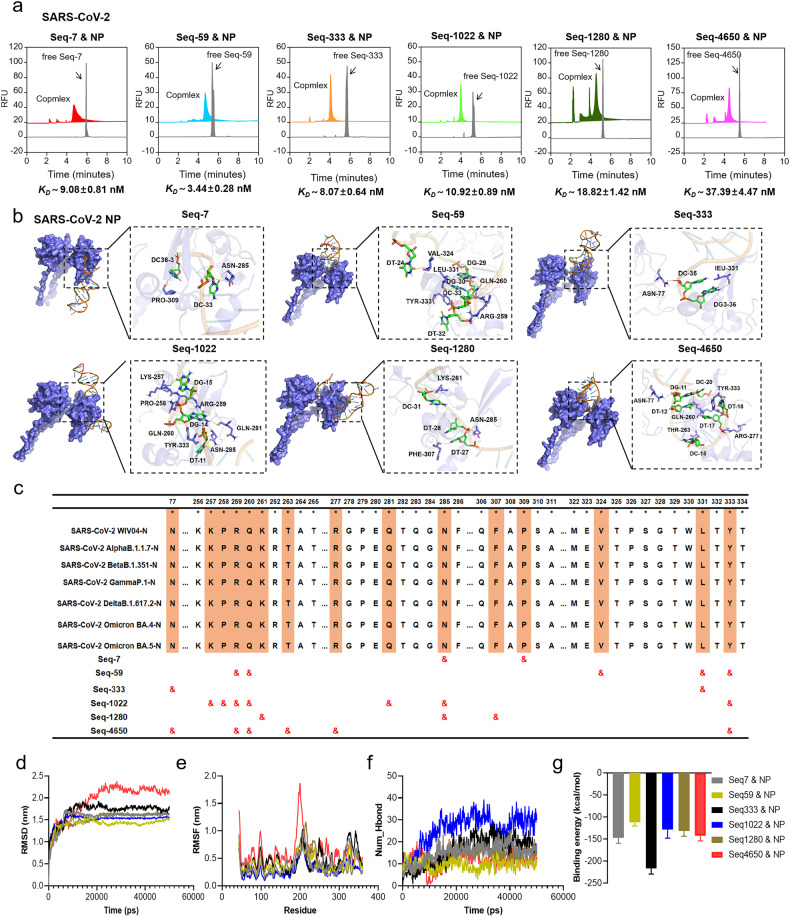
Characterization of the affinity and the Binding sites prediction between aptamers and N protein of SARS-CoV-2. **a** Affinity and specificity characterizations between aptamers and N protein of SARS-CoV-2 were performed via CE, *n* = 3 per group. **b** The results of molecular docking to predict and simulate the binding sites of aptamers to SARS-CoV-2 NP using Discovery Studio software. The NP is shown with residue names and identifier. **c** The binding sites of six ssDNA aptamers to NP in different SARS-CoV-2 mutants. **d**–**g** Molecular dynamics simulation results between aptamers and NP of SARS-CoV-2. **d** Root mean square deviation (RMSD). **e** Root mean square fluctuation (RMSF). **f** Number of intermolecular hydrogen bonding (H-bonds). **g** The binding energy of aptamers and NP of SARS-CoV-2

Based on the characteristics that the affinity of specific binding is higher than that of non-specific binding, we designed the following screening scheme (Fig. 1a): 1) N protein immobilized magnetic prepared, 2) mixing of magnetic bead-coupled N protein with nucleic acid pool for affinity enrichment screening, 3) polymerase chain reaction (PCR) amplification, 4) detecting with polyacrylamide gel electrophoresis (PAGE) and analyzing with next-generation sequencing. The obtained enriched libraries are then identified for affinity, when it is reached as follows: 1) An increase in the retention rate of positive screening, a decrease in the retention rate of reverse screening, and a significantly higher retention rate in positive screening compared to reverse screening (Fig. 1b); 2) Affinity of the library was determined using surface plasmon resonance (SPR), with the pool showing stable binding of > 90 RU, indicating significant enrichment and suitability for sequencing (Fig. 1c).

The precise binding of nucleic acids to target proteins is essential in aptamer screening, which involves repetitive elution and binding procedures to enrich nucleic acids with affinity for target proteins. However, repeated elution and binding stages may result in non-specific binding by nucleic acids due to non-sequence-dependent interactions. To avoid this behavior, we optimized the screening process by considering secondary structure and variant clustering. Taking into account the secondary structure required to support interactions between biomolecules, such as neck loops and hairpin structures can play an important role in determining the specificity of molecular interactions, and specifically binding depends on contact between a few key sites, we were able to eliminate sequence-independent determined affinity behavior. Therefore, we selected the top 10,000 nucleic acid molecules in the pool 5 as screening objects, and further analyzed them using cluster analysis and structural entropy screening. This process ultimately resulted in the identification of 165 potential aptamer molecules (Fig. 1d). Then, combined with antiviral experiments, we screened 38 aptamer molecules with inhibitory activity compared to the virus control group (CT ≤ 14.59) (Supplementary Table 1). Further, by combining these features with the frequency ranking of aptamer screening pool 5, we identify 6 aptamers with intact neck loops and hairpins using secondary structure simulation (Fig. 1d–f).

The affinity of the six selected aptamers with the N protein of SARS-CoV-2 was measured via capillary electrophoresis (CE) and SPR experiment. CE chromatogram shows the binding status and intensity of coronavirus NPs and aptamers. The peak of free NP aptamers appeared from 5.5 min to 6.0 min, after interacting with coronavirus NPs, free aptamers peak decreases or disappears, while complex peaks of monomorphism or polymorphism appear at 2.0 ~ 5.0 min, which indicated that NP aptamers bind strongly to coronavirus NPs and form stable complexes. The complexes formed by coronavirus NPs and NP aptamers presented differentiated CE characteristic chromatograms. The complexes formed by NP aptamers and SARS-CoV-2 NP have various charge-mass ratios, and they are separated during capillary zone electrophoresis, showing polymorphic complex peaks (Fig. 2a), which may be caused by different binding ratios. Further, we quantitatively evaluate the affinity of NP aptamers to coronavirus NPs. Equilibrium dissociation constant (K_D_) and Binging percent in Supplementary Fig. 1, which indicated that NP aptamers have low nanomolar K_D_ values, and their affinity to SARS-CoV-2 NP and SARS-CoV NP is higher than that of MERS NP, -OC43 NP and -NL63 NP. The results showed that all six aptamers obtained from the SARS-CoV-2 inhibition screening exhibited high affinity to the SARS-CoV-2 N protein, with K_D_ values ranging from 3.44 to 37.39 nM (Fig. 2a, Supplementary Fig. 1). The K_D_ value obtained by SPR was shown in Supplementary Fig. 2. The K_D_ value measured by CE is lower than that measured by SPR, which may be due to the need to immobilize the target protein during the determination of binding process by SPR, resulting in masking of some protein domains. Additionally, molecular docking revealed the specific binding interactions between the aptamers and certain amino acid residues of the N protein. Seq-7 was found to bind with ASN-285 and PRO-309 in the CTD region of the N protein, Seq-59 exhibited binding with ARG-259, GLN-260, VAL-324, LEU-331, and TYR-333 residues in the CTD domain. Seq-333 interacted with ASN-77 in NTD domain and LEU-331 residues in CTD, Seq-1022 formed interactions with LYS-257, PRO-258, ARG-259, GLN-260, GLN-281, ASN-285, and TYR-333 residues, Seq-1280 bound to LYS-261, ASN-285, and PHE-307 residues, and Seq-4650 interacted with ASN-77 in NTD and ARG-259, GLN-260, THR-263, ARG-277, and TYR-333 residues in CTD (Fig. 2b). Importantly, the binding sites of these aptamers to SARS-CoV-2 N protein exhibited 100% conservation across all different SARS-CoV-2 variants and partial results were shown in Fig. 2c which provides a crucial structural basis for the inhibitory effects of the aptamers against different SARS-CoV-2 variants. The results of our molecular dynamics (MD) simulations clearly demonstrated that within systems involving aptamers and target complexes, all aptamers effectively establish stable interactions with the SARS-CoV-2 N protein (Fig. 2d–g). However, it is noteworthy that the complex formed by Seq-4650 and the SARS-CoV-2 N protein exhibited suboptimal characteristics. This suboptimal behavior is substantiated by pronounced fluctuations in Root Mean Square Deviation (RMSD) and Root Mean Square Fluctuation (RMSF), a reduced number of hydrogen bonds, and lower binding energy. On the other hand, when examining the complex systems involving the SARS-CoV-2 N protein and aptamers Seq-7, Seq-59, Seq-333, Seq-1022, and Seq-1280, our observations reveal that Seq-7, Seq-1022, and Seq-1028, upon complexity with the SARS-CoV-2 N protein, exhibit superior performance in terms of RMSD, RMSF, hydrogen bond count, and binding energy compared to the complexes formed with Seq-59 and Seq-333. As a result, these complexes demonstrate enhanced protein hydrophobicity and a more stable tertiary structure.

### The uptake efficiencies and internalization mechanism exploration of ssDNA aptamer

**Fig. 3:**
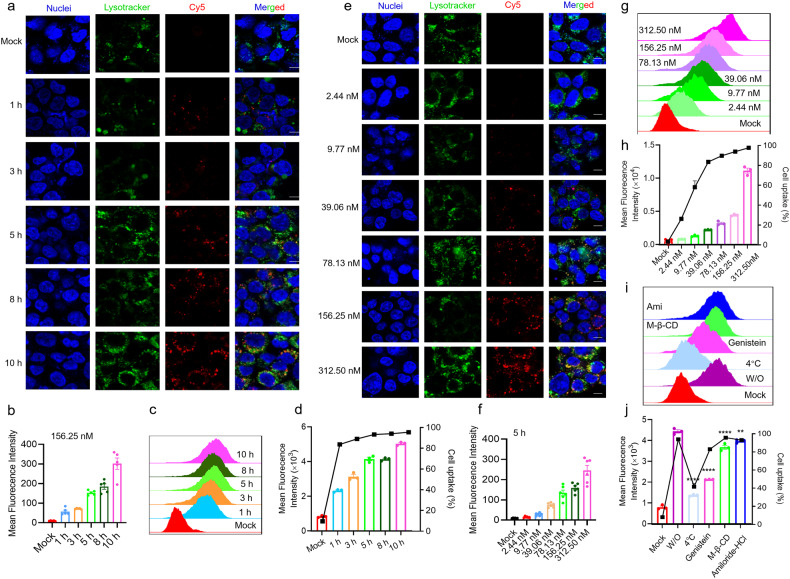
The uptake efficiencies and internalization mechanism exploration of ssDNA in HEK-293T cell. **a** CLSM was applied to observe the subcellular localization and intracellular intensity of Cy5-labeled ssDNA aptamers (156.25 nM) in HEK-293T cells at different treatment time points. Scale bars, 10 μm. **b** Mean fluorescence intensities (MFIs) of Cy5-labeled aptamers in HEK-293T cells related to (**a**). **c** The uptake efficiencies of Cy5-labeled aptamers (150 nM) at different treatment time points were evaluated by flow cytometry. **d** MFIs recorded in (**c**). **e** The subcellular localization and intracellular intensity of Cy5-labeled aptamers in HEK-293T cells was observed at different concentrations after 5 h of treatment via CLSM, scale bars, 10 μm. **f** Quantitative analysis of (**e**). **g** The uptake efficiencies of Cy5-labeled aptamers at different concentrations after 5 h treatment was evaluated by flow cytometry. **h** Quantitative analysis of (**g**, **i**) Some specific endocytosis inhibitors were applied to explore the internalization mechanism of Cy5-labeled aptamers (150 nM). **j** Relative MFI and cell uptake rate of Cy5-labeled aptamers recorded in (**i**), *n* = 3 per group, all data are shown as the mean ± SEM, Statistical analyses were done using the univariate analysis of variance (ANOVA) with SPSS 21.0 statistical software. Where “**” represents *P* < 0.01, “****” represents *P* < 0.0001. vs the W/O group

When SARS-CoV-2 enters the host cell and undergoes genome replication, the N protein will be exposed, making it accessible for aptamers to bind and consequently inhibit the virus proliferation. However, the ability of the aptamer to enter host cells is a critical step for its potential efficacy. Therefore, we carefully investigated the internalization and intracellular trafficking of ssDNA aptamer into HEK-293T cells and 16HBE cells at different treatment time points. Aptamer Seq-1022 with 36-nucleotide labeled with Cy5 fluorescence (Cy5-ssDNA) was applied and the confocal laser scanning microscopy (CLSM) data revealed that as the treatment time was extended, the Cy5 fluorescence intensity in the cell increased independently, indicating continuous internalization of Cy5-ssDNA by cells (Fig. 3a, b; Supplementary Fig. 3), and 5 h after treatment, more aptamers had entered the cell, which was in line with the results of Fluorescence-activated cell sorting (FACS) (Fig. 3c, d). Moreover, CLSM and FACS were also used to observe the subcellular localization and intracellular intensity of Cy5-ssDNA aptamers in HEK-293T cells at different concentrations after 5 h of treatment. It was observed that the higher concentration of ssDNA aptamers, the more aptamers entered the cells in a dose-dependent manner, leading to an accumulation of aptamers in HEK-293T and 16HBE cells (Fig. 3e, f; Supplementary Fig. 3). The FACS data were consistent with the above results (Fig. 3g, h; Supplementary Fig. 3).

In addition, to explore the mechanism of endocytosis, some specific endocytosis inhibitors including amiloride-HCl (Amil), Methyl-β-cyclodextrin (M-β-CD), genistein (Geni), and 4 °C were used to block macropinocytosis, lipid rafts-mediated endocytosis, caveolae-mediated endocytosis and energy-mediated endocytosis, respectively. Compared with W/O group transfected without inhibitors, the uptake efficiency of cells pretreated with Amil, M-β-CD or genistein inhibitors was observed that the cellular entry of the Cy5-ssDNA was slightly inhibited (Fig. 3i, j), indicated that aptamer can enter cells through various pathways, including macropinocytosis, lipid rafts-mediated endocytosis, caveolae-mediated endocytosis, and energy-mediated endocytosis. Additionally, the co-localization signal of Cy5 with lysosomes indicates that a significant proportion of aptamers does not co-localize with lysosomes (Fig. 3a, e; Supplementary Fig. 3b), suggesting that aptamers possess a degree of lysosomal escape capability.

### Antiviral activity of the aptamers against SARS-CoV-2 Prototype and omicron

**Fig. 4:**
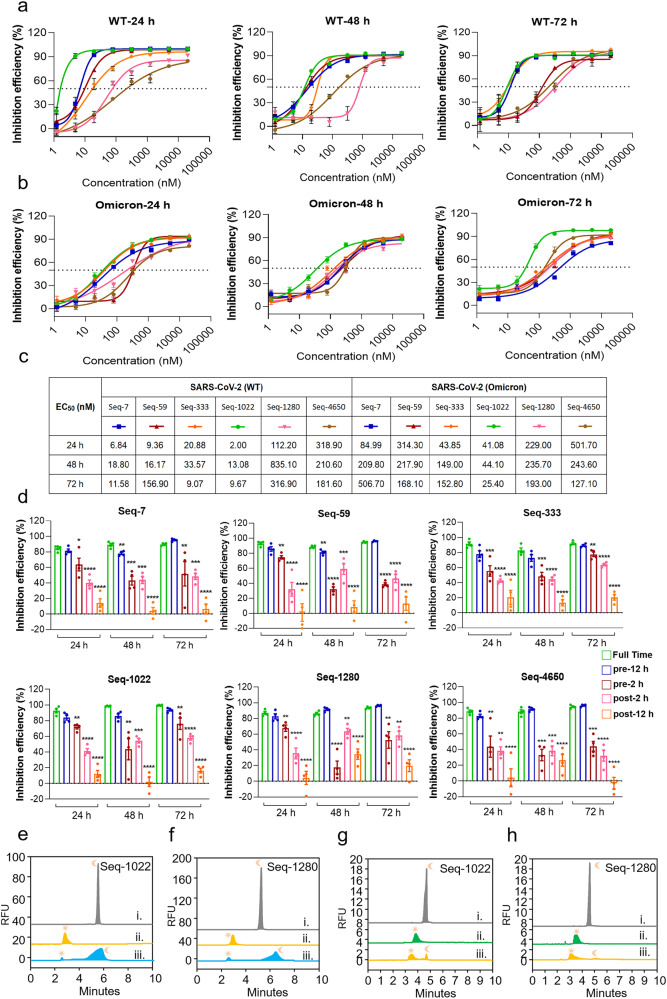
The antiviral activities of the six aptamers against SARS-CoV-2 prototype and Omicron in vitro **a** Inhibition efficiency of authentic SARS-CoV-2 prototype and (**b**) Omicron (BA.5) via ssDNA aptamers at 24 h, 48 h and 72 h post of infection. EC_50_ values were calculated using the competitive enzyme-linked immunosorbent assay (ELISA) standard curve fitting (GraphPad Prism 8.0 Software, La Jolla, CA, USA) and presented in (**c**). **d** Time-of-addition experiment of six aptamers, the concentration of aptamers was 10 µM, *n* = 4 per group. All data are shown as the mean ± SEM, Statistical analyses were done using the univariate analysis of variance (ANOVA) with SPSS 21.0 statistical software. where “*” represents *p* < 0.05; “**” represents *p* < 0.01; “***” represents *p* < 0.005; “****” represents *p* < 0.0001. vs the Full-time group. **e** CE analysis of competitive binding interactions between polyundylic acid and Seq-1022 with SARS-CoV-2 NP, i. 500 nM Seq-1022; ii. 500 nM Seq-1022 + 1000 nM SARS-CoV-2 NP; iii. 500 nM Seq-1022 + 1000 nM SARS-CoV-2 NP + 3.3 mg/mL polyundylic acid. “☾” represents the free aptamer; “☀” represents the complex of aptamer and SARS-CoV-2 NP. **f** CE analysis of competitive binding interactions between polyundylic acid and Seq-1280 with SARS-CoV-2 NP, i. 500 nM Seq-1280; ii. 500 nM Seq-1280 + 1000 nM SARS-CoV-2 NP; iii. 500 nM Seq-1280 + 1000 nM SARS-CoV-2 NP + 3.3 mg/mL polyundylic acid. **g** CE analysis of competitive binding interactions between anti-SARS-CoV-2 NP Ig G and Seq-1022 with SARS-CoV-2 NP. i. 100 nM Seq-1022; ii. 100 nM Seq-1022 + 200 nM SARS-CoV-2 N; iii. 100 nM Seq-1022 + 200 nM SARS-CoV-2 NP + 200 nM antiNP Ig G. “☾” represents the free aptamer; “☀” represents the complex of aptamer and SARS-CoV-2 NP. **h** CE analysis of competitive binding interactions between anti-SARS-CoV-2 NP Ig G and Seq-1280 with SARS-CoV-2 NP. i. 100 nM Seq-1280; ii. 100 nM Seq-1280 + 200 nM SARS-CoV-2 N; iii. 100 nM Seq-1280 + 200 nM SARS-CoV-2 NP + 200 nM antiNP Ig G

We further investigated the inhibitory capacity of aptamers (Seq-7, Seq-59, Seq-333, Seq-1022, Seq-1280, and Seq-4650) against the authentic virus of SARS-CoV-2 prototype and its Omicron variant to better understand their antiviral ability. The aptamers mentioned above successfully inhibited both the SARS-CoV-2 prototype and its omicron variants. We selected the aptamer CoV2-6C3, which has been previously shown strong anti-SARS-CoV-2 activity in vitro and a random sequence as our experimental control (Supplementary Fig. 4). Seq-1022 showed significant anti-SARS-CoV-2 prototype ability with EC_50_ values of 2.00 nM, 13.08 nM, and 9.67 nM at 24 h, 48 h, and 72 h post-infection, respectively (Fig. 4a). Seq-7, Seq-59, and Seq-333 similarly demonstrated potent antiviral activity against the SARS-CoV-2 prototype 24 h post-infection, with the EC_50_ value of 6.84, 9.36 and 20.88 nM, respectively. However, Seq-59 showed less persistence with EC_50_ values of 9.36 nM at 24 h-infection and 156.90 nM at 72 h post-infection. Meanwhile, the inhibitory ability of Seq-7 and Seq-59 were lessened by the viral mutation, with corresponding 24, 48, and 72 h post-infection, the EC_50_ values ranging from 6.84 nM to 84.99 nM for Seq-7 against SARS-CoV-2 prototype and 84.99 nM to 506.70 nM against omicron (BA.5) (Fig. 4b). Interestingly, Seq-1022 showed comparable inhibition against both the SARS-CoV-2 prototype and the omicron variant. The EC_50_ values of Seq-1022 for the prototype were 2.00 nM, 13.08 nM, and 9.67 nM at 24, 48, and 72 h post-infection, respectively, while for the omicron variant were 41.08 nM, 44.10 nM, and 25.40 nM at 24, 48, and 72 h post-infection, respectively. The positive control, CoV2-6C3 also showed significant antiviral activity with EC_50_ values of 69.23 to 177.10 nM against SRS-CoV-2 prototype at 24 h, 48 h, and 72 h post-infection (Supplementary Fig. 4). It is noteworthy that CoV2-6C3 demonstrates a notable decrease in its inhibitory efficacy against the Omicron variant, with its EC_50_ value peaking at 498.6 nM. This diminished activity is attributed to the fact that CoV2-6C3 is an aptamer designed to target the receptor binding domain (RBD) of the SARS-CoV-2 spike protein. Given the substantial mutations have accrued in the RBD of spike protein during viral evolution, this has resulted in a marked reduction in the effectiveness of CoV2-6C3. This highlights the increased significance and urgency of developing aptamers that target the N protein. We then carried out further validation to assess the efficacy of the aptamers in preventing and treating SARS-CoV-2 infection, using a full-course drug additive group as a positive control. The aptamers demonstrated promising effects, particularly in the pre-infection scenario. In particular, when Seq-1022 was administered 12 h before SARS-CoV-2 Omicron strain infection, it exhibited inhibition rates of 83.75%, 86%, and 93.5% after 24 h, 48 h, and 72 h of authentic virus infection, respectively. The inhibition rate for the full-time addition group was 92.5%, 98.5%, and 99.5%, respectively (Fig. 4c). Other data also showed that aptamer agents were more effective when administered before the infection than post-infection, likely because it takes time for the aptamers to enter the host cell. Notably, administering aptamers 12 h prior to infection was as effective as full-course drug addition. These findings support the potential of aptamers as promising prophylactic antiviral agents for COVID-19. Furthermore, in order to assess the adaptability of our antiviral candidates, we performed infection experiments using the HCoV-OC43 virus. The results showed that the aptamers displayed a concentration-dependent inhibitory effect on HCoV-OC43 (Supplementary Fig. 5). However, it is worth noting that the EC_50_ value for HCoV-OC43 exhibited a significant increase compared to SARS-CoV-2 and its variants.

The main function of the N protein is to assemble the gRNA into the gRNA-N protein complex, also known as vRNP, during the final stage of the virus life cycle. To provide a more comprehensive understanding of the antiviral mechanism of the aptamers, we carried out competitive binding experiments between aptamers and a viral RNA analog, polyundecylic acid targeting the N protein using CE. These experiments aimed to determine whether the aptamers possess the capability to impede viral replication by competitively binding to the N protein, potentially disrupting its interaction with viral RNA. We labeled FAM on aptamers and established a ternary competitive binding system consisting of aptamers, viral RNA analogues, and SARS-CoV-2 NP. As shown in Fig. 4d, free Seq-1022 peaked at 5.80 min (spectral line a). After adding SARS-CoV-2 NP at twice of the Seq-1022 concentration, all the free Seq-1022 were bound to form the aptamer-SARS-CoV-2 NP complex at 3.06 min (line b.). Further, 3.3 mg/mL polyundylic acid was introduced into the competing system, and it was found that the peak of the aptamer-SARS-CoV-2 NP complex decreased, and the free Seq-1022 peak reappeared (line c). Due to the introduction of high concentration of polyundylic acid into the competitive system, a significant competitive binding reaction occurs, and the aptamer-SARS-CoV-2 NP complex is greatly reduced. This data confirmed that the aptamer can compete with viral RNA analogs in binding SARS-CoV-2 NP, indicating that the aptamer has the ability to produce inhibitory effects on SARS-CoV-2 NP binding viral RNA. Similarly, Seq-1280 also showed competition with polyundylic acid for the binding of SARS-CoV-2 NP (Fig. 4e). Similarly, we also found Seq-1022 and Seq-1280 could form a binding competition with anti-SARS-CoV-2 NP Ig G against SARS-CoV-2 NP (Fig. 4f, g). Several studies have suggested that the NP of HCoVs may trigger cellular apoptosis,^32–34^ which is in line with our results. Additionally, we further demonstrated that Seq-1022 could effectively hinder apoptosis induced by NP in 16HBE cells by interfering with its functionality (Supplementary Fig. 6).

In terms of safety, all aptamers demonstrated excellent biocompatibility and exhibited no apparent cytotoxicity in different cell types even at a concentration of 50 μM in vitro (Supplementary Figs. 7, 8). The stability of aptamers, both in vitro and in vivo, plays a critical role in their practical applications. To assess this, we conducted an evaluation of aptamer stability by incubating them in cell media containing 10% FBS at 37 °C for different times. The results revealed that when exposed to an environment containing 10% FBS, aptamer Seq-1022 exhibits remarkable stability. Following a 72 h storage period at 37 °C, we observed no significant degradation of the aptamer, as confirmed by both agarose and PAGE gel electrophoresis (Supplementary Fig. 9a, b). This observation elucidates the sustained potent antiviral activity of our aptamers in vitro, even after a 72 h incubation period. In our animal model studies, we simulated the application scenario of the aptamer through intratracheal injection. This approach allowed for sustained Cy5 fluorescence expression in the lungs for a period of 72 h (Supplementary Fig. 9c). Both in vitro and in vivo experiments demonstrated that the relevant aptamer exhibits a good stability.

### High affinity of aptamers for binding with other known HCoVs

**Fig. 5:**
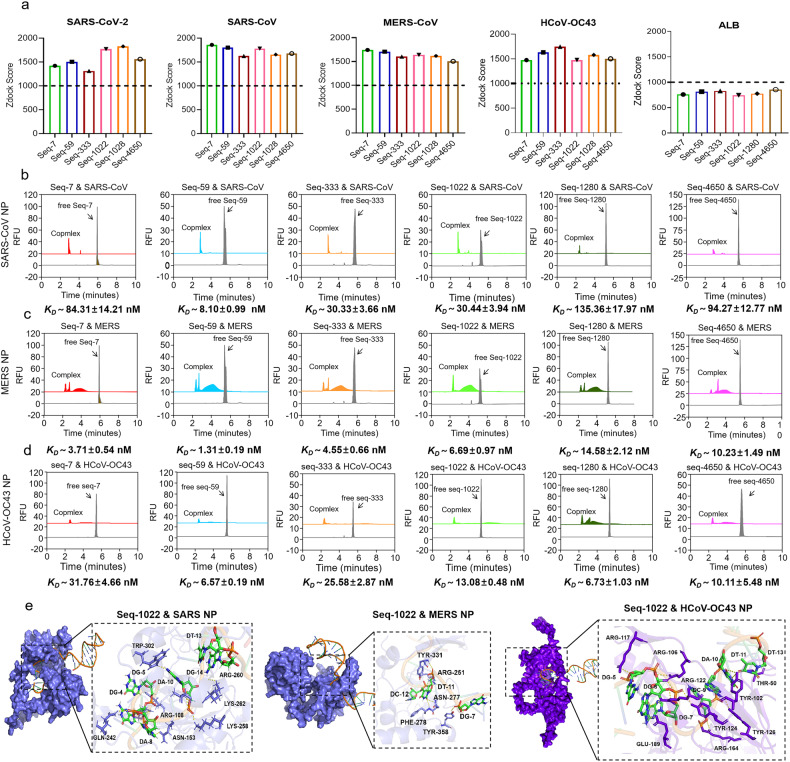
Prediction of binding and affinity identification between the aptamers and the N proteins of SARS, MERS and -OC43. **a** Prediction of the binding affinity between the aptamers and N proteins of SARS, MERS and HCoV-OC43 via Zdock score. The N protein of SARS-CoV-2 and ALB protein were used as positive and negative control, respectively. The characterizations of affinity and specificity between aptamers and N proteins of (**b**) SARS, (**c**) MERS and (**d**) -OC43 were performed via CE. **e** The results of molecular docking to predict and simulate the binding sites of aptamer Seq-1022 to N proteins of SARS, MERS and -OC43 using Discovery Studio software

The effects of aptamers on HCoVs of SARS, MERS, -OC43 and -NL63 were further evaluated. First, we used software simulation scores to evaluate the binding of the aptamers to the N proteins of SARS, MERS, -OC43 and -NL63, SARS-CoV-2 NP and human serum albumin (ALB) were used as positive and negative control, respectively (Fig. 5a, Supplementary Fig. 10). A zdock score higher than 1000 indicates a high likelihood of binding, with higher values indicating better binding affinity. These aptamers demonstrate a favorable binding affinity with the N proteins of SARS, MERS, -OC43 and -NL63, with some of them exhibiting even higher affinity than the N protein of SARS-CoV-2. We further confirmed the affinity using CE. The complex of aptamers and SARS-CoV NP showed a single sharp complex peak (Fig. 5b), indicating that the complex had the same charge-mass ratio and a fixed binding ratio. Aptamers combined with MERS NP to form 3 ~ 4 stable complex peaks (Fig. 5c), including 2~ 3 sharp complex peaks (2.0 ~ 3.0 min) and a dispersion peak (~4.0 min). The complexes of -OC43 (Fig. 5d) and -NL63 NP (Supplementary Fig. 10) with aptamers are similar to SARS-CoV NP, but -NL63 is a dispersion dissociation region formed by unbound NP aptamers from 3.1 min to 5.8 min. SPR analysis confirms CE results that NP aptamers have broad spectrum binding to coronavirus NPs (Supplementary Fig. 11). It is noteworthy that differentiated complex characteristic chromatograms can provide support for aptamer identification of different kinds of coronavirus NPs. All data of K_D_ value and Binging percent evaluated by CE were shown in Supplementary Fig 1. The results showed that all six aptamers exhibited high affinity to the four N proteins, with K_D_ values ranging from 8.10 to 135.36 nM in SARS NP, 1.31–14.58 nM in MERS NP, 6.57–31.76 nM in -OC43 NP and 3.24–24.18 nM in -NL63 NP, respectively (Fig. 5b–d, Supplementary Fig. 1 and Supplementary Fig. 10). Subsequently, molecular docking was applied to simulate the binding sites of the aptamers to the N proteins of SARS, MERS and -NL63 (Fig. 5e, Supplementary Figs. 12–15). Among them, Seq-1022 interacted with ARG-108, ASN-153, GLN-242, LYS-258, ARG-260 and TRP-302 residues in NP of SARS and ARG-251, ASN-277, PHE-278, TYR-331 and TYR-358 residues in NP of MERS. ARG-259 in the NP of SARS-CoV-2, ARG-260 in the NP of SARS, and ARG-251 in the NP of MERS are highly conserved amino acids throughout evolution which can be recognized by aptamer Seq-1022 (Figs. 2b, 5e, Supplementary Figs. 12, 13, and 15). Additionally, these aptamers exhibited good affinity for N proteins of other HCoVs (-229E, and -HKU1) based on protein docking methods (Supplementary Fig. 16). Among them, the antiviral activity of aptamers against HCoV-OC43 was verified (Supplementary Fig. 5). Through the protein docking analysis, we firmly believe that the corresponding aptamer agents could also effectively inhibit viral replication in SARS, MERS and even other unknown coronavirus infections.

## Discussion

The N protein of SARS-CoV-2 consists of 419 amino acids and shares structural features with other human coronaviruses (HCoVs). It is characterized by two conserved domains: the N-terminal domain (NTD, aa:44-174) and the C-terminal domain (CTD, aa: 255-364). Additionally, it possesses three intrinsically disordered regions (LDR): the N-arm, flexible linker region (LKR), and C-tail.^35^ The functions of coronavirus N proteins can be broadly categorized into two major roles. Firstly, they play a crucial role in assembling the genomic RNA (gRNA) into the gRNA-N protein complex, also known as the viral ribonucleoprotein (vRNP), during the final stages of the virus life cycle. Secondly, N proteins contribute to the suppression of the host cell’s immune response and manipulation of cellular machinery to enhance viral replication.^36–38^ Given these critical functions, the N protein’s conservation is paramount, not only in SARS-CoV-2 variants but also across various human coronaviruses. This conservation ensures the proper binding of N proteins with diverse nucleic acids, allowing only those HCoVs with functional N proteins to survive and propagate during evolution.

The N-terminal domain (NTD) of CoVs N protein serves as an RNA-binding domain, interacting with a broad spectrum of viral and host cell nucleic acids, encompassing both RNA and DNA.^39^ Within the NTD, a protruding β-hairpin is predominantly comprised of basic amino acid residues, generating a positively charged pocket at the junction between the core structure and the basic hairpin. This configuration forms an RNA binding site.^39^ Previous investigations indicate that both single-stranded RNA (ssRNA) and double-stranded RNA (dsRNA) exhibit similar binding to the positively charged pocket of the NTD. Arginine residues (R92, R107, and R149) directly engage with the RNA in this binding process. The C-terminal domain (CTD) plays a role in dimerization/oligomerization, forming high-order structures.^40^ Recent findings have revealed that the CTD exhibits an even greater affinity for binding nucleic acids compared to the NTD. It demonstrates a heightened affinity for the 32-mer stem-loop II motif (S2m) derived from the SARS-CoV-2 genome.^41^ The N-CTD dimer also establishes a positively charged pocket groove on the helix face^21,42,43^ featuring highly conserved amino acid residues (K256, K257, R259, K261, and R262) (Supplementary Fig. 15). These residues are crucial for CTD’s ability to bind viral RNA.^21,35,42,43^ In our study, all six aptamers were found to recognize and bind to N-CTD. Among them, Seq-1022, Seq-59, Seq-1280, and Seq-4650 could recognize and bind to the conserved amino acid residues from K257 to K261, potentially disrupting the function of the positively charged pocket groove of the CTD, which is essential for binding to viral RNA. However, despite the absence of amino acid mutations in the binding sites of the aptamers with NP, the inhibitory effect of the aptamers on the Omicron strain was slightly diminished compared to that on prototype (Fig. 4a, b). This observation could be attributed to the spatial structural changes in the Omicron N protein.

Another pivotal attribute of the HCoVs N-CTD is its capacity to form various oligomers, including dimers, trimers, tetramers, or hexamers, in a concentration-dependent manner. In a solution environment, the CTD of the SARS-CoV-2 NP presents a compact homodimeric structure characterized by an overall rectangular slab shape. Previous studies have elucidated the dimerization of the N protein, wherein N-CTD dimers engage with two N-terminal domains (N-NTDs) through the disordered linker regions (LKRs), resembling two arms. This property is crucial for the self-association of N protein, facilitating viral genome encapsidation. Consequently, targeting the normal oligomerization of the N protein or inducing aberrant formation of the ribonucleoprotein (RNP) complex emerges as an attractive inhibitory strategy. Recently, Lin et al. identified 5-benzyloxygramine (P3) as a novel MERS inhibitor through virtual screening.^44^ This compound has the potential to promote non-native dimerization of MERS N-NTD and induce aggregation of N proteins. For other viruses, such as human immunodeficiency virus^45^ and influenza virus,^46^ researchers have proposed inhibiting viral N protein oligomerization by developing competitive peptides. For HCoVs, studies have demonstrated that peptides targeted to the C-terminal tail sequence interfere with the CTD oligomerization of HCoV-229E N protein, resulting in decreased viral titer.^47^ This provides a relevant reference for studies on the SARS-CoV-2 N protein. In the current study, the binding of the six aptamers to N-CTD has the potential to disrupt the function of the N protein by interrupting its self-association.

These aptamers could be considered as potential drugs for combating SARS-CoV-2, raising concerns regarding its stability, administration method, and dosage. In our in vitro experiments, we observed the remarkable stability of these aptamers, enduring for up to 72 h in a cell culture serum environment. This stability can be attributed to their intricate secondary structures and abundant A/T complementary sequences, which significantly enhance the thermodynamic stability of nucleic acids.^48,49^ During the initial aptamer screening using SELEX, nonspecific binding is excluded, and specific binding is enriched, allowing direct analysis of binding specificity from the screening frequency ranking. Considering the stability of aptamers, we also consider the spatial structure of aptamers as a crucial factor in the early screening process.^50^ The complex spatial structure of aptamers, such as G-quadruplexes and i-motifs, contributes to forming more intricate spatial structures, enhancing their specificity toward target proteins.^51–53^ Structures like loops,^54^ and pseudoknots^55^ are essential functional groups that relate to the high affinity activity of aptamers. The structural characteristics and stability of our aptamers are used as auxiliary screening indicators to conduct preliminary screening of SELEX results, which is crucial for experimental efficiency.^56^ Dosing routes and administration regimens can vary dramatically between aptamers based on their design and intended use. For example, aptamer-REG1, targeting Coagulation factor IX-binding protein subunit A (IXa),^57,58^ and the ARC1779^59,60^ targeting willebrand factor, are primarily administered intravenously. In contrast, aptamers like Macugen,^61,62^ AS1411,^63,64^ E10030,^65,66^ ARC1905,^67^ used to treat macular degeneration, are administered intravitreally. For anti-SARS-CoV-2 aptamer drugs, nasal spray may be a viable option due to its efficient respiratory delivery route. Our in vivo experiments revealed that when administered through intratracheal injection, the Cy5 signal of aptamer predominantly concentrates in the lungs and remains detectable for an impressive duration of up to 72 h. This provides assurance for the potential applications of our aptamers.

To our knowledge, this is the first study to evaluate the antiviral activity of DNA aptamers targeting the N protein of SARS-CoV-2. Previous studies have primarily focused on the antiviral effects of aptamers targeting the S protein, which can block virus entry into host cells. However, as mutations accumulate in the S protein of SARS-CoV-2, the neutralizing activity of these aptamers may gradually decline. Therefore, the aptamers targeting N protein in our study and those targeting the Spike protein could be used as a cocktail therapy, which is expected to improve the antiviral effects by both blocking the process of viral entry and inhibiting viral replication. Due to the evolutionary conservation of the N protein, inhibitors targeting the N protein theoretically could have activity against a broad range of coronaviruses. However, these drugs would need to enter cells to be effective, which rules out the direct application of antibody-based drugs. Small molecule drugs or nucleic acid-based drugs might be more likely to exert their function and should be a focus of attention.

Nevertheless, the study also has several limitations. Firstly, our assessment of aptamers focused solely on their in vitro antiviral activity. To comprehensively understand their potential, additional studies on in vivo antiviral activity is imperative, given the complex environment where diverse factors may affect aptamers’ antiviral efficacy. Evaluating the stability and homogeneity of aptamers in vivo is also essential and warrants further investigation. In addition, a thorough exploration of aptamer toxicity through in vivo studies is necessary to fully harness their potential as antiviral agents. In fact, current research on the in vivo application of aptamer drugs still faces numerous challenges. Some challenges of aptamers in the development of antiviral drugs may be addressed through formulation with novel materials or delivery by nano particles, which needs further evaluation.

In summary, we identified six DNA aptamers with high affinity to the N protein of SARS-CoV-2, which showed potent viral inhibition activity against both the prototype strain and the Omicron (BA.5) variant. Furthermore, our results suggest that these aptamers may have anti-pan-coronavirus capability, as indicated by protein docking analysis, the affinity detection and anti-HCoV-OC43 activity. These findings provide an important foundation for the development of aptamer-based therapeutics against life-threatening infections.

## Materials and methods

### Materials

H_3_BO_3_, Na_2_B_4_O_7,_ and NaOH were purchased from Aladdin (Shanghai, China). Polyundecylic acid (Poly (U)) was purchased from Sigma-Aldrich (P9528). The single-stranded DNA (ssDNA) library contains a 40-base random region flanked by two 20-base primer regions (5’-FAM-AGC AGC ACA GAG GTC AGA TG-(N40)-CCT ATG CGT GCT ACC GTG AA-3’). The 5’ terminal was labeled with 6-carboxyfluorescein (FAM). The forward and reverse primer sequences are as follows: P: 5’-AGC AGC ACA GAG GTC AGA TG-3’, P’: 5’-CCT ATG CGT GCT ACC GTG AA-3’, respectively. The fluorescently labeled ssDNA was purified by reversed-phase HPLC. All DNA libraries, PCR primers, and aptamers were obtained from Sangon Biotech (Shanghai) Co., Ltd. SARS-CoV-2 NP was purchased from Sangon Biotech (D140006-0001). SARS NP (40143-V08B), MERS NP (40068-V08B), RBD of spike protein of SARS-CoV-2 Omicron strain (40592-V08H121) and SARS-CoV-2 Nucleocapsid monoclonal antibody (40588-R0004) were purchased from Sino Biological Inc. Normal human serum was obtained from Beijing BioDee Biotechnology Co., Ltd. Human albumin was obtained from Sigma-Aldrich (St. Louis, MO). Luciferase Assay System and Passive Lysis Buffer (PLB) were bought from Promega Co. Ltd. (Madison, USA). Lysotracker Green DND-26 and DAPI were obtained from Sigma-Aldrich. Cell Counting Kit (CCK-8) (40203ES76) and Annexin V-FITC/PI Apoptosis Detection Kit (40302ES60) were bought from Yeasen Biotechnology (Shanghai) Co., Ltd. The cell culture reagents, including trypsin, penicillin-streptomycin, Opti-MEM, Dulbecco’s modified Eagle’s medium (DMEM), fetal bovine serum (FBS) and Lipofectamine 2000 were purchased from Thermo Fisher Scientific Inc.

Unless otherwise noted, all samples and buffers were prepared in ultrapure water.

### Magnetic bead-based -SELEX

We conducted DNA-SELEX utilizing the WT N protein of SARS-CoV-2 as the target. The oligonucleotide library, sourced from Sangon Biotech, consists of 40 random nucleotides flanked by constant primer sequences. Magnetic bead-SELEX was employed for the screening process. Initially, 5 nmol of ssDNA library was dissolved in a 500 μL binding buffer (PBS with 0.55 mM MgCl_2_), annealed at 95 °C, immediately cooled on ice for 5 min, and then at 25 °C for 10 min. The N protein was conjugated with carboxyl magnetic beads following the carboxyl magnetic beads kit protocol (Sangon Biotech). After a 30 min incubation of N-beads with the library at 25 °C, the supernatant was removed, and the recovered beads underwent two washes in a binding buffer. Subsequently, the recovered N-beads were introduced into a PCR mixture (comprising a forward primer, biotin-labeled reverse primer, dNTPs, Taq DNA polymerase, and PCR buffer) to amplify the target-bound sequences. For the generation of a single-stranded library, the PCR product was incubated with streptavidin-coated sepharose beads (GE Healthcare) and denatured using 0.1 M NaOH for 1 min. Following desalting with 3 K ultrafiltration tubes (Millipore), the product served as the library in the subsequent round. Simultaneously, the selected library underwent amplification through the PCR (Supplementary Table 2) and was assessed by 8–10% native polyacrylamide gel electrophoresis (PAGE). Eventually, the library was analyzed using next-generation sequencing.

### Cell lines and viruses

HEK-293T cell (ATCC, CRL-3216), HEK-293T cell stably expressing ACE2 (HEK293T-ACE2) (Vazyme Biotech Co., Ltd.), African green monkey kidney Vero cells (ATCC, CCL-81), Human bronchial epidermal cells (16HBE, ATCC), Baby Hamster Kidney Fibroblasts cells (BHK21, ATCC) were cultured in Dulbecco’s minimal essential medium (DMEM) (Gibco) supplemented with 10% fetal bovine serum (FBS) (Corning) and penicillin (10,000 U/mL)-streptomycin (10 mg/mL) (Gibco). To evaluate the impact of aptamers on SARS-CoV-2 and HCoV-OC43 replication, Vero cells, HEK-293T, or BHK21 cells were infected with the SARS-CoV-2 prototype strain (Beta/Shenzhen/SZTH-003/2020, GISAID accession number: EPI_ISL_406594), Omicron BA.5 strain (SZTH40-P2.1-7, Genome Warehouse accession number: GWHBMBF01000000), and HCoV-OC43 strain (GenBank accession number AY391777.1) at a multiplicity of infection (MOI) of 0.01, followed by treatment with varying aptamer concentrations. Viral replication was assessed at 24 h, 48 h, and 72 h post-infection using quantitative reverse transcription polymerase chain reaction (qRT-PCR). All SARS-CoV-2 and HCoV-OC43 infection procedures adhered to biosafety level 3 and biosafety level 2 laboratory standards, respectively, at Shenzhen Third People’s Hospital, following an approved protocol. The sequence of the RBD aptamer (CoV2-6C3) is 5’-CGC AGC ACC CAA GAA CAA GGA CTG CTT AGG ATT GCG ATA GGT TCG G, and the sequence of the random control is 5’-TGA TTG AGT GAC GCA GCA TGG ACA CGG TGG CAA CAG.

Time-of-addition experiment of six aptamers: For “Full-time” treatment, HEK293T-ACE2 cells were pre-treated with aptamers for 12 h, and virus was added to allow attachment for 2 h. Afterwards, the virus-drug mixture was removed and the cells were cultured with aptamer-containing medium until the end of the experiment. For “pre-12 h” treatment, HEK293T-ACE2 cells were pre-treated with aptamers for 12 h, and the medium containing aptamer was discarded before virus added. At 2 h p.i., the medium containing virus was replaced by fresh culture medium until the end of the experiment. For pre-2 h treatment, the aptamers were added into cells for 2 h before viral attachment, the virus-aptamer mixture was replaced with fresh medium at 2 h p.i. and maintained till the end of the experiment. For post-2 h group, aptamer-containing medium was added into cells after 2 h of virus infection. For post-12 h group, aptamer-containing medium was added to replace the fresh medium after 12 h of virus infection.

### Molecular docking

The 3D structures of N proteins from SARS, SARS-CoV-2, MERS, -229E, -HKU1, -OC43, and -NL63 were retrieved from the RCSB Protein Data Bank (http://www.rcsb.org). PyMOL 2.4.0 (open-source version) was utilized to eliminate the original ligand, solvent molecules, redundant protein chains, and polar hydrogen atoms. The 3D structure of the ssDNA aptamer was initially generated using RNA composer, which was then converted into DNA 3D coordinates through the Mode RNA webserver and MDWeb webserver. The docking box for the N protein was predicted based on literature and the UniProt knowledge base database. Subsequently, ZDOCK was employed for molecular docking to assess melatonin binding and the hub targets. All sampled binding modes underwent evaluation using the iterative knowledge-based scoring function ITScorePP. Ultimately, the binding modes were ranked according to their binding energy scores, and the top ten binding modes were reported. Default parameters were maintained throughout the docking calculation, and the docking results were visualized using PyMol 2.4.0 software.

### Gromacs molecular dynamics simulation verification

Based on molecular docking, we substantiated our findings through molecular dynamics (MD) simulation. MD simulation serves as a computational method for mimicking the movement of small molecules within a biological environment. Gromacs software was employed for the MD simulation, setting the physical conditions to a constant temperature (300 K), constant pressure (101 kPa), and periodic boundary conditions. The protein water model was utilized to replicate the human environment in a neutral sodium chloride solution of 0.145 mol/L. Following the equilibration of all environmental states, a 50 ns MD simulation was conducted for the screened compound-target complex system. During this simulation, confirmation storage calculations were performed at intervals of 100 ps, the analysis encompassed Root Mean Square Deviation (RMSD), Root Mean Square Fluctuation (RMSF), and the number of intermolecular hydrogen bonds (H-bonds), all analyzed using Gromacs. The binding energy was computed using gmx_MMPBSA.

### Affinity determination via capillary electrophoresis (CE)

#### Co-incubation of NP protein and NP Aptamers

To achieve the desired conformation of NP Aptamers, a 100 μM solution of NP Aptamers underwent heat treatment at 94 °C for 5 min, followed by a gradual cooling to 4 °C before application. Complex formation involved the precise mixing of volumes from NP Aptamers and NP proteins stock solutions, subsequently diluted with incubation buffer to attain the target concentrations of 200 nM for NP Aptamers and 100 nM for NP proteins. The final sample solution volume was 10 µL. Prior to injection into CE, the sample solution was incubated at 37 °C for 10 min.

#### Capillary electrophoresis conditions

The separation and detection of NP proteins and NP aptamers mixtures were carried out using a Beckman P/ACE MDQ system (Beckman-Coulter, Fullerton, CA, USA) equipped with a LIF (Laser-Induced Fluorescence) detector, enabling the visualization of FAM-labeled NP aptamers. Analysis of all CE data was performed using 32 Karat software. Separation was achieved using an uncoated fused silica capillary (75 μm i.d. × 50.2 cm (40.0 cm effective), Sino Sumtech, Handan, Hebei, China) at a constant temperature of 25 °C. Sample injection into the capillary was conducted at a pressure of 0.5 psi for 10 s, and separation was driven by a running voltage of 20 kV (498 V/cm). Excitation was generated using the 488 nm line of an Ar+ laser (Beckman Coulter), with emission collected at 520 nm. The running buffer consisted of 50 mM H_3_BO_3_/Na_2_B_4_O_7_ (pH 7.8). To mitigate potential adsorption of NP proteins on the capillary’s inner surface and ensure reproducible CE separation, the capillaries underwent a treatment sequence involving 0.1 M NaOH for 3 min, followed by water for 3 min, and running buffer for an additional 3 min after each consecutive sample injection.

### Affinity determination via surface plasmon resonance (SPR)

The affinity between aptamers and NPs of different HCoVs was assessed using surface plasmon resonance (SPR), with the RBD of the spike protein from the SARS-CoV-2 Omicron strain serving as a control. Briefly, NPs in acetate buffer (pH 5.5) was injected into the sensor chip to achieve immobilization at a level of -1000 RU. Deactivation was carried out using ethanolamine-HCl to block unreacted carboxyl groups. Binding analysis between the N protein and aptamers at different concentrations was conducted using a Biacore T200 instrument (GE Healthcare). A running buffer composed of PBS and 0.005% tween-20 was used, while 50 mM NaOH served as the regeneration buffer. Sensorgrams, capturing the association/dissociation behavior of the aptamer-NP or aptamer-RP complex upon injection of aptamers, were collected. Data analysis was performed using the 1:1 Langmuir model provided in the BIAevaluation software (version 4.1) to calculate the equilibrium dissociation constant K_D_.

### Cellular uptake and mechanism

The cellular uptake of Cy5-labeled aptamers was assessed in HEK-293T cells. Initially, 1 × 10^5^ HEK-293T cells were seeded in plates and incubated overnight. Subsequently, the standard DMEM was replaced with Opti-MEM medium, and the cells were transfected with aptamers at various concentrations for 5 h or with 150 nM aptamers over different durations. The Cy5-fluorescence signal was then detected using a FACS Calibur flow cytometer (BD, USA) and/or a Confocal laser scanning microscope (CLSM). Quantitative analysis of mean fluorescence intensities was conducted using Flowjo 7.6.1 or Nikon NIS-Elements analysis software.

To elucidate the endocytosis mechanism of aptamers in HEK-293T cells, specific inhibitors (100 µM amiloride-HCl (Amil), 5 mM Methyl-β-cyclodextrin (M-β-CD), 1 mM genistein (Geni), and 4 degrees) were employed. Initially, the endocytosis inhibitors were added to the cell culture medium 1 h before treatment. Following this, 150 nM Cy5-ssDNA aptamers were introduced to each group, and the cells were cultured for 5 h at 37 °C. Inhibitor-free cells were considered a positive control, and after 5 h of aptamer treatment, the cells were analyzed using the FACS Calibur flow cytometer (BD, USA).

### Cell viability

Cell viability was tested using the Cell Counting Kit-8 (CCK-8) assay. Briefly, HEK-293T cells were initially seeded in a 96-well plate (10,000 cells per well) and incubated overnight in a 5% CO_2_ humidified atmosphere. Subsequently, the cells were exposed to various concentrations of aptamers (0.078125, 0.15625, 0.3125, 0.625, 1.25, 2.5, 5, 10, 50 µM) for 24 h. The mock group consisted of cells treated without aptamers. Following incubation, 10 µL of CCK-8 solution was added to each well, and the plate was further incubated at 37 °C for 2–4 h. Absorbance at 450 nm was measured using a multimode microplate reader. Cell viability in each group was calculated by normalizing to untreated cells.

### Cell apoptosis

The 16HBE cells were seeded into a 12-well plate (1 × 10^5^ cells per well) and cultured overnight in a humidified atmosphere containing 5% CO_2_. Afterward, the standard DMEM in each well was replaced with 1 mL of Opti-MEM media, and the cells were transfected with plasmids using Lipofectamine 2000 for a 6 h period. The plasmids expressed N proteins of SARS, MERS, and SARS-CoV-2, with a transfection concentration of 1 μg/mL. Following transfection, 150 nM aptamers were introduced to the cells for a duration of 5 h. Finally, cell apoptosis was evaluated after 24 h of aptamer treatment using the Annexin V-FITC/PI Apoptosis Detection Kit, and the analysis was performed with a FACS Calibur flow cytometer (BD, USA).

### Analysis of the stability of aptamer

#### In vitro stability assessment

To evaluate the stability of aptamer in vitro, 10 μM aptamer was incubated in DMEM containing 10% FBS at 37 °C for 72 h. Samples were collected at 0, 0.5, 1, 2, 4, 8, 24, 48, 72 h, then stored at −20 °C. Subsequently, aptamer separation was achieved through 5% agarose gel electrophoresis or 20% polyacrylamide gel electrophoresis (PAGE) at 120 V. Gel visualization was performed using a UV imaging system (Tanon, Shanghai, China), and post-electrophoresis analysis was carried out with a molecular imager (BIO-RAD).

#### In vivo biodistribution

To evaluate the distribution and metabolism of aptamers in vivo, Balb/c mice were divided into two groups, receiving either PBS or Cy5-labeled Seq-1022 (1 mg/kg) via intratracheal injection (i.t.). Whole-body Cy5 fluorescence signals were detected at 1, 3, 6, 9, 24, 48, and 72 h post-injection using the Kodak in vivo imaging system FX Pro (Carestream Health, USA). Additionally, one animal was sacrificed at each time point in both groups, and tissues (heart, lung, liver, spleen, and kidney) were isolated for signal examination with the Kodak in vivo imaging system FX Pro. All the animal experiments in our study were approved and carried out in accordance with the principles of the Institutional Animal Care and Use Committee of Beijing Institute of Technology.

### Statistical analysis

Significance analysis was performed by GraphPad Prism 8.0 software. All of the data are presented as the mean ± SEM. Statistical difference was analyzed by univariate analysis of variance (ANOVA) or Student’s t-test with SPSS 21.0 statistical software. Where “*” represents *p* < 0.05; “**” represents *p* < 0.01; “***” represents *p* < 0.005; “****” represents *p* < 0.001. EC_50_ values were calculated using the competitive ELISA standard curve fitting (GraphPad Prism 8.0 Software, La Jolla, CA, USA).

### Supplementary information

Supplementary Materials

## Data Availability

All data supporting the findings of this study are available within the article and its Supplementary Information files. Raw data is available from the corresponding authors upon request.
