# Supplementary material for: Aptamers targeting SARS-CoV-2 nucleocapsid protein exhibit potential anti pan-coronavirus activity
Source: Signal Transduct Target Ther. 2024 Feb 14;9:40. doi: 10.1038/s41392-024-01748-w (PMC10866937; doi:10.1038/s41392-024-01748-w)
Supplement: Supplementary file 1 — Supplementary Materials [file 41392_2024_1748_MOESM1_ESM.docx]

**Supplementary Materials for**

Aptamers targeting SARS-CoV-2 nucleocapsid protein exhibited potential anti pan-coronavirus activity

Minghui Yang^1†^, Chunhui Li^1†^, Guoguo Ye^2†^, Chenguang Shen^3^, Huiping Shi^1^, Liping Zhong^5^, Yuxin Tian^1^, Mengyuan Zhao^1^, Pengfei Wu^1^, Abid Hussain^1^, Tian Zhang^1^, Haiyin Yang^1^, Jun Yang^1^, Yuhua Weng^1^, Xinyue Liu^1^, Zhimin Wang^1^, Lu Gan^5^, Qianyu Zhang^5^, Yingxia Liu^2^, Ge Yang^4*^, Yuanyu Huang^1*^, Yongxiang Zhao^5*^

Correspondence to: [yongxiang_zhao@126.com](mailto:yongxiang_zhao@126.com), [yyhuang@bit.edu.cn](mailto:yyhuang@bit.edu.cn) or [yangge@imb.cams.cn](mailto:yangge@imb.cams.cn).

**This PDF file includes:**

Supplementary Figure 1-16 with their legends

Supplementary Table 1- 2

**Supplementary Figures and Figure Legends**


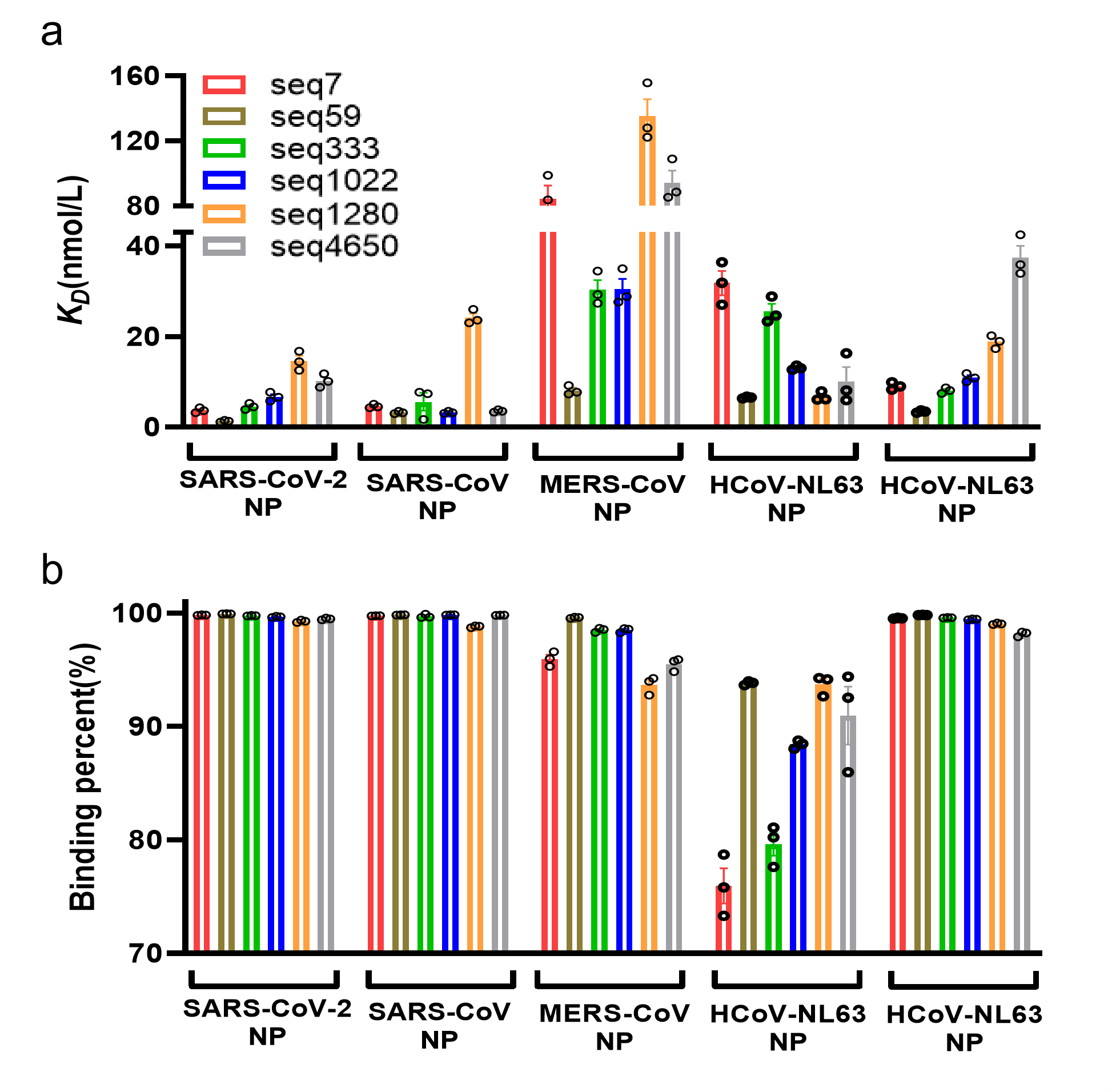


**Supplementary Figure 1. The *K_D_* (a) and Binging percent (b) of NP aptamers to Coronavirus NPs evaluated by Capillary Electrophoresis.**


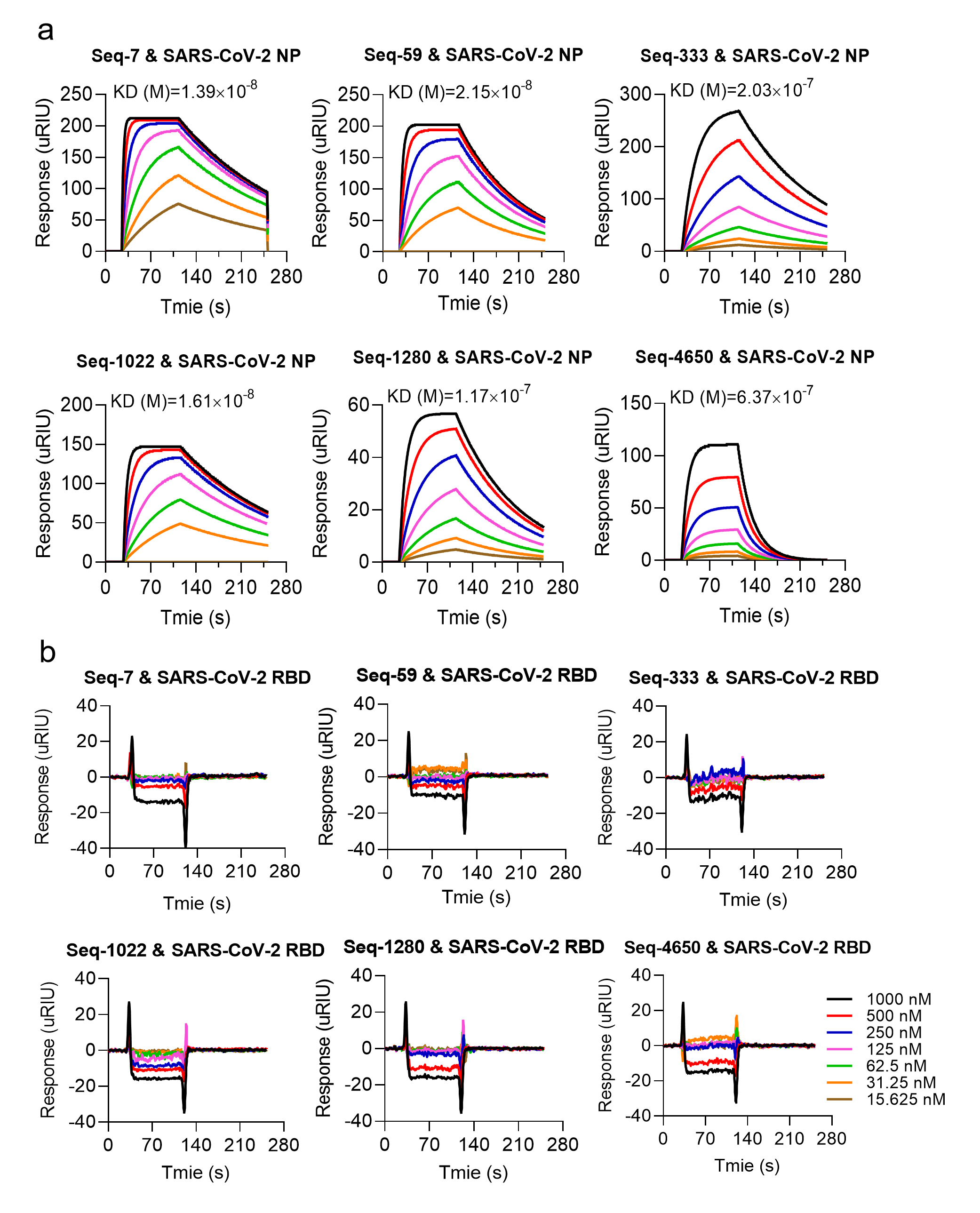


**Supplementary Figure 2. Characterization of the affinity** between six aptamers and **(a)** NP of SARS-CoV-2 via SPR and **(b)** the RBD protein was introduced as a negative control.

**
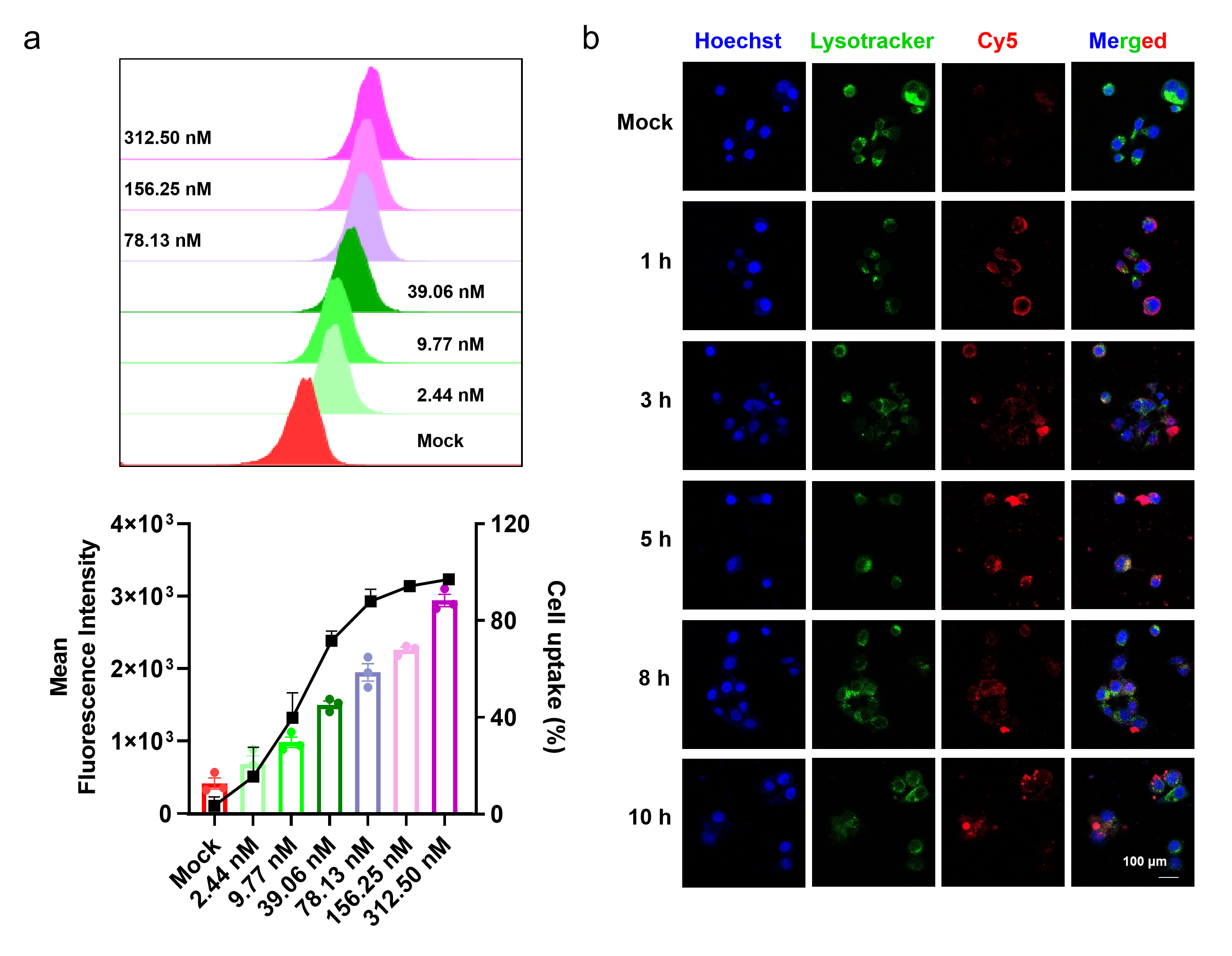
**

**Supplementary Figure 3.** **The uptake/internalization kinetics and efficiencies of ssDNA in 16HBE cells.** **(a)** Transfection efficiencies of Cy5-labeled ssDNA aptamers at different transfection concentrations after 5 h transfection was evaluated by flow cytometry and quantitative analysis. All data are shown as the mean ± SEM, n=3 per group. **(b)** CLSM was applied to observe the subcellular localization and intracellular intensity of Cy5-labeled ssDNA aptamers (150 nM) in 16HBE cells at different transfection time points. Scale bars, 100 μm.

**
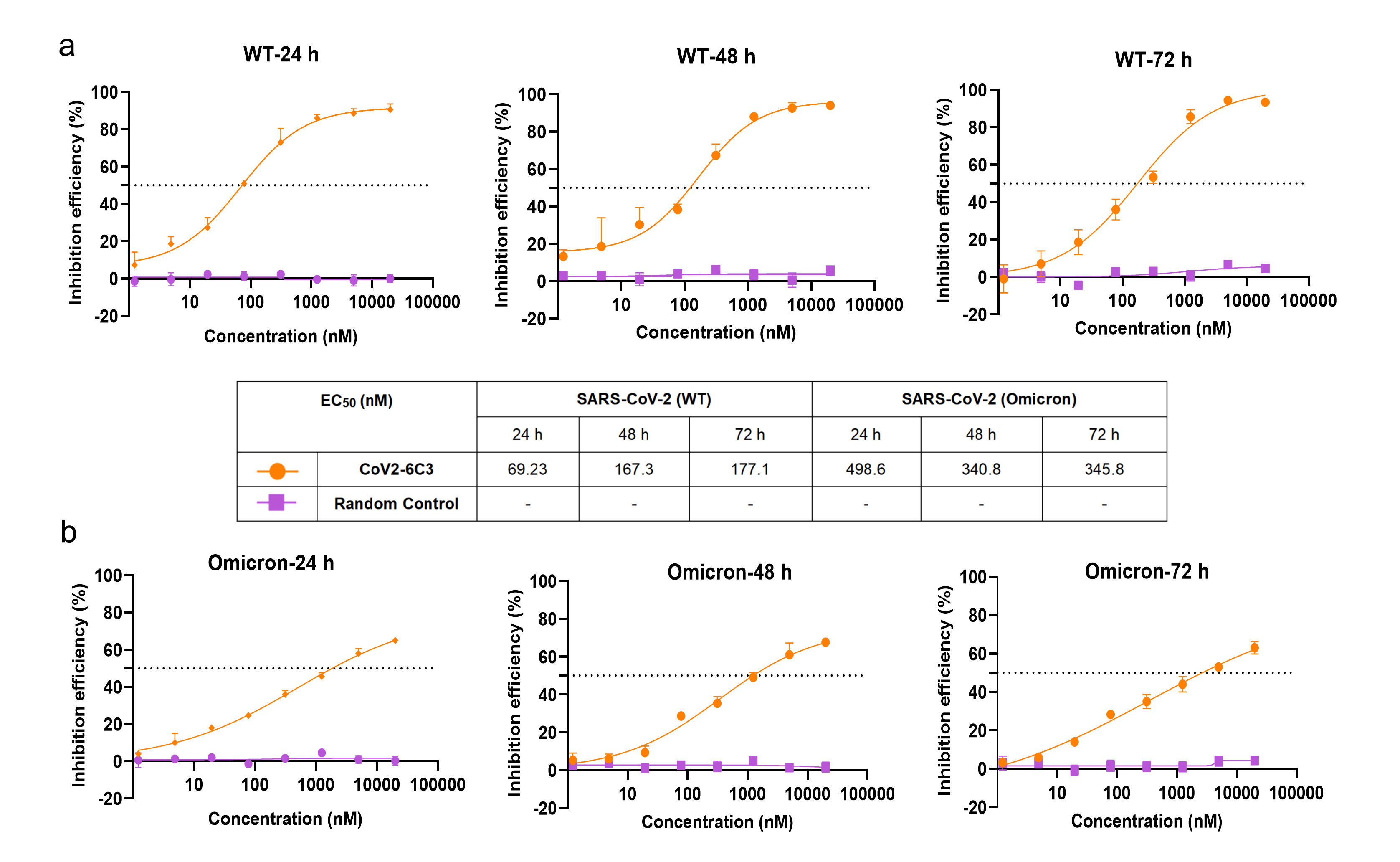
**

**Supplementary Figure 4.** **The antiviral activities of positive (CoV2-6C3) and negtive (Random) control against SARS-CoV-2 prototype (a) and Omicron BA.5 (b) in vitro.** The EC_50_ values were calculated using the competitive ELISA standard curve fitting (GraphPad Prism 8.0 Software, La Jolla, CA, USA) and the results are succinctly summarized in the central table.


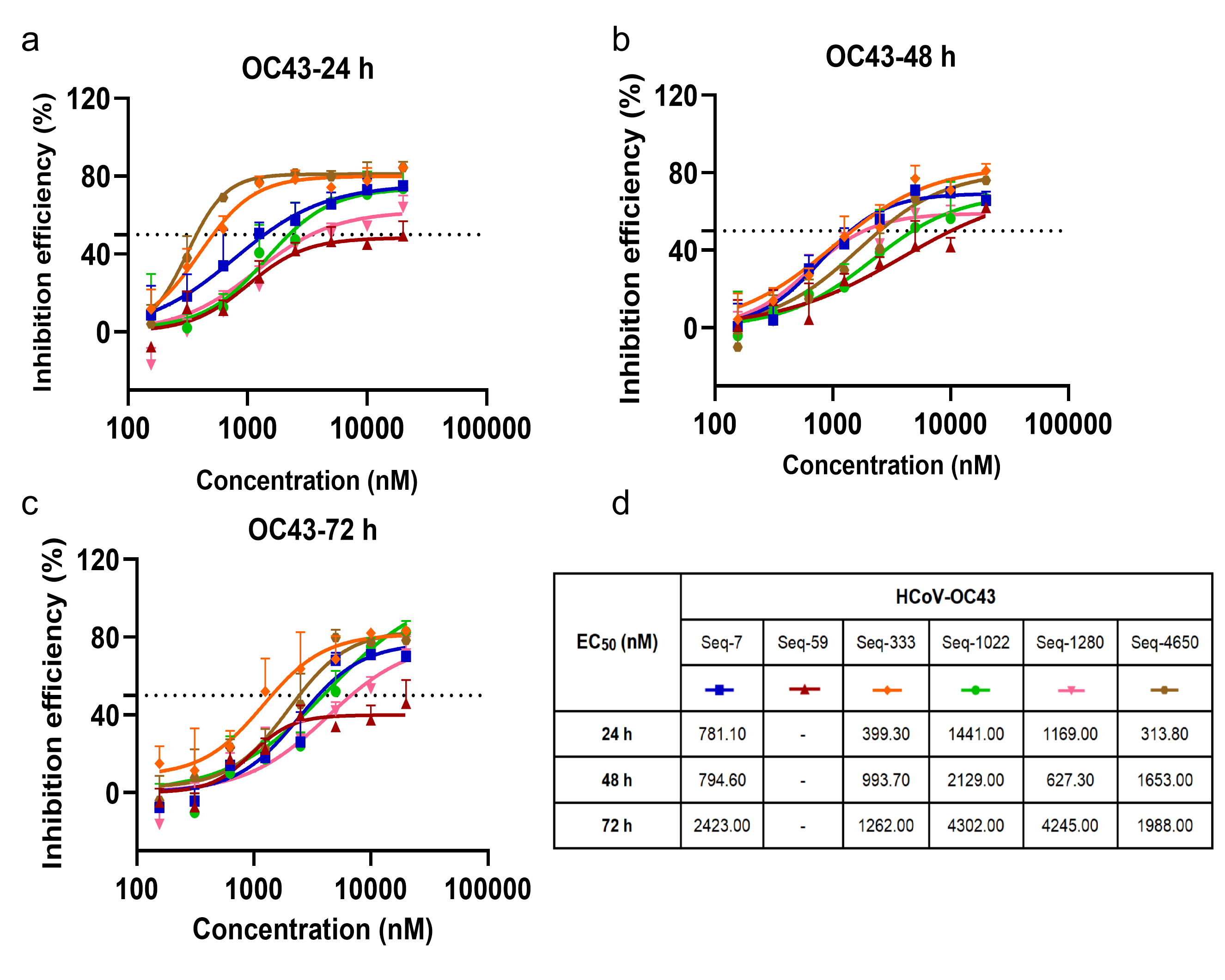


**Supplementary Figure 5.** **The antiviral activities of six aptamers against HCoV-OC43 at 24 h (a), 48 h (b) and 72 h (c) post of infection *in vitro*.** The EC_50_ values were calculated using the competitive ELISA standard curve fitting (GraphPad Prism 8.0 Software, La Jolla, CA, USA) and presented in **(d)**.


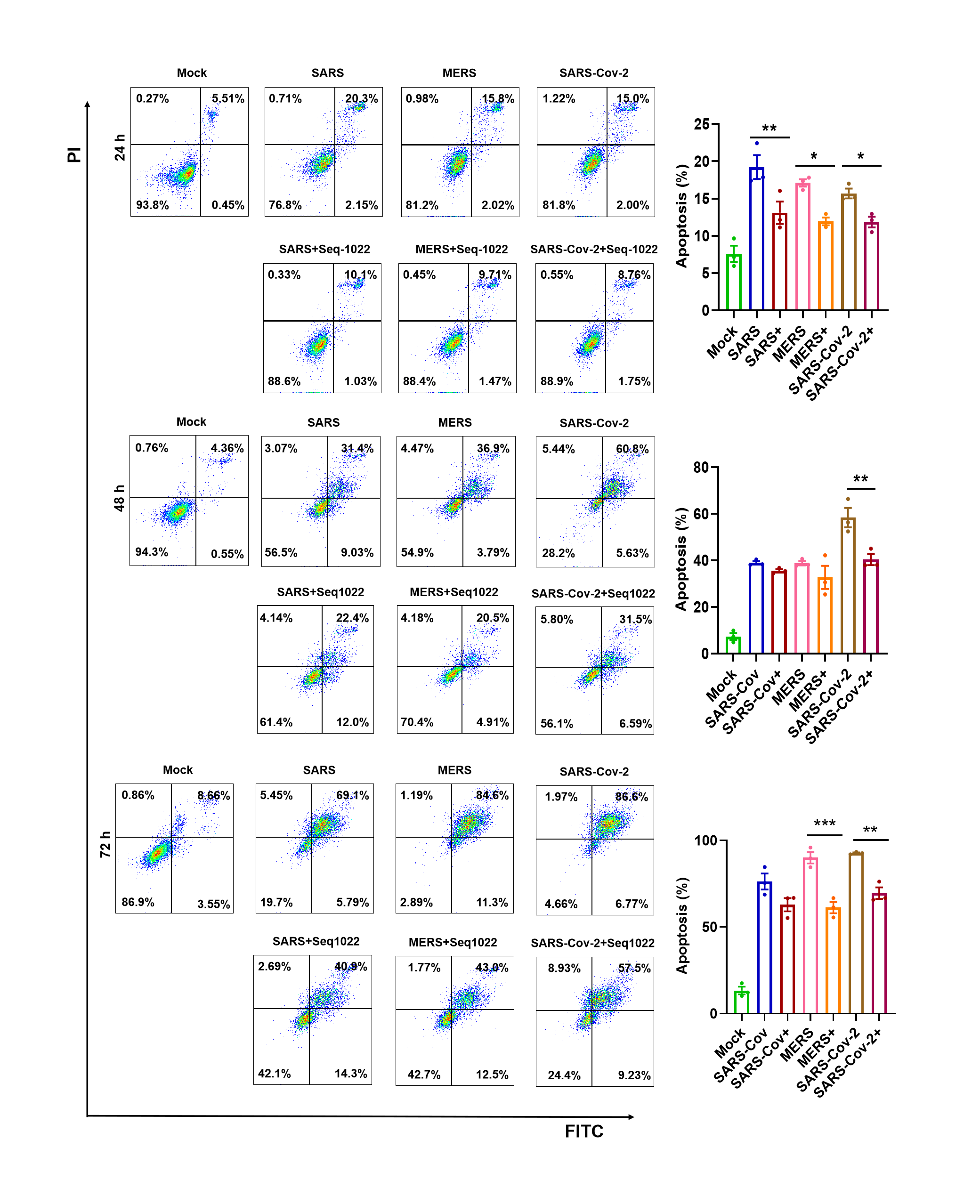


**Supplementary Figure 6.** **Effects of aptamer Seq-1022 on N protein-induced cell apoptosis in 16HBE cells at 24 h, 48 h and 72 h post transfection.** The DNA plasmids of MERS-CoV-N, SARS-CoV-2-N, SARS-CoV-N were transfected into 16HBE cells to assess their apoptotic effects. The designations of MERS+, SARS-CoV-2+, and SARS-CoV+ corresponded to cells transfected with the DNA plasmids and subsequently treated with the Seq-1022 (150 nM), n=3 per group. All of the data are presented as the mean ± SEM. Statistical difference was analyzed by Student's t-test, where “*” represents p < 0.05; “**” represents p < 0.01; “***” represents p < 0.005.


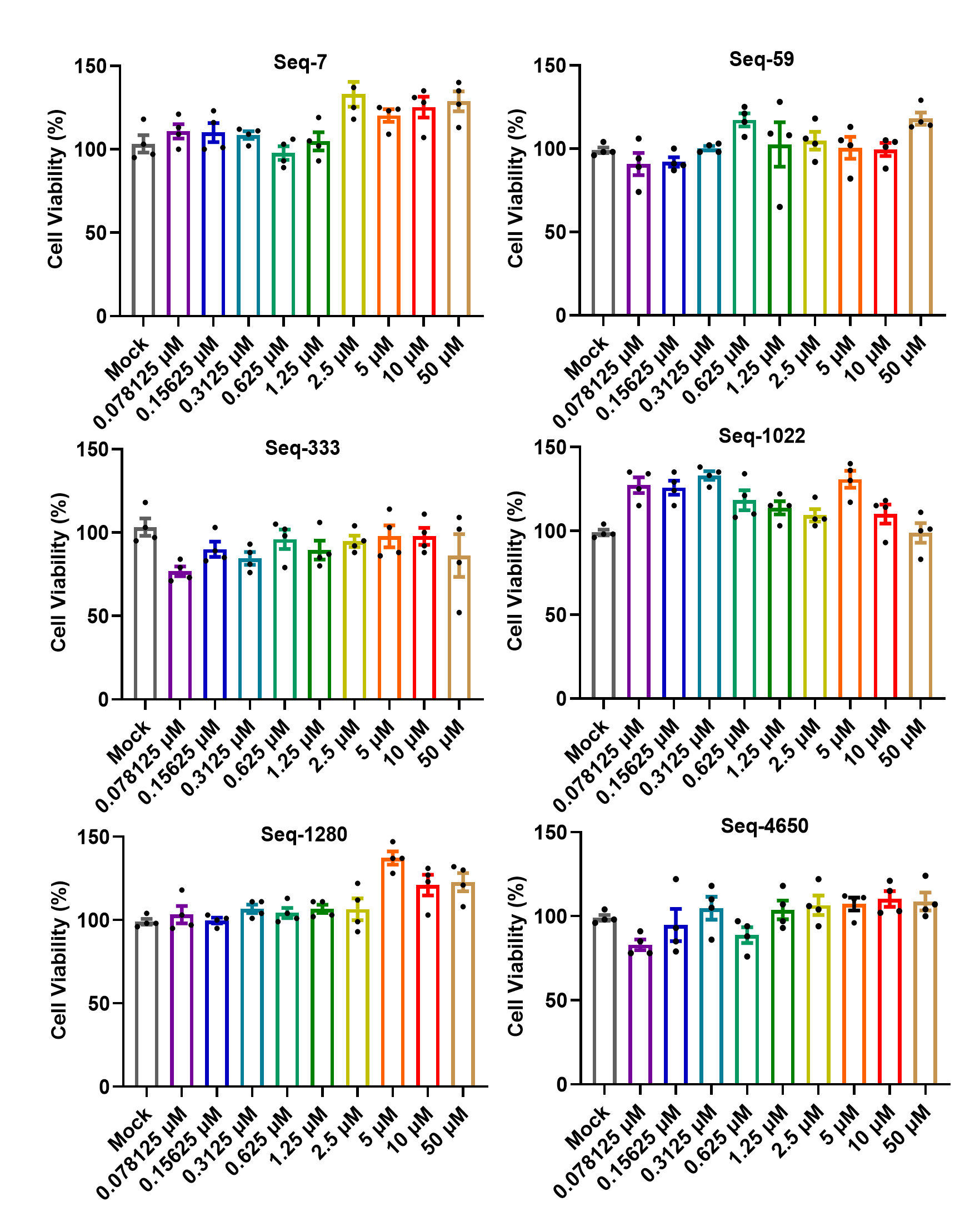


**Supplementary Figure 7.** **Cytotoxicity of six aptamers evaluated by CCK-8 in HEK293 cells.** n=3 per group, all data are shown as the mean ± SEM.


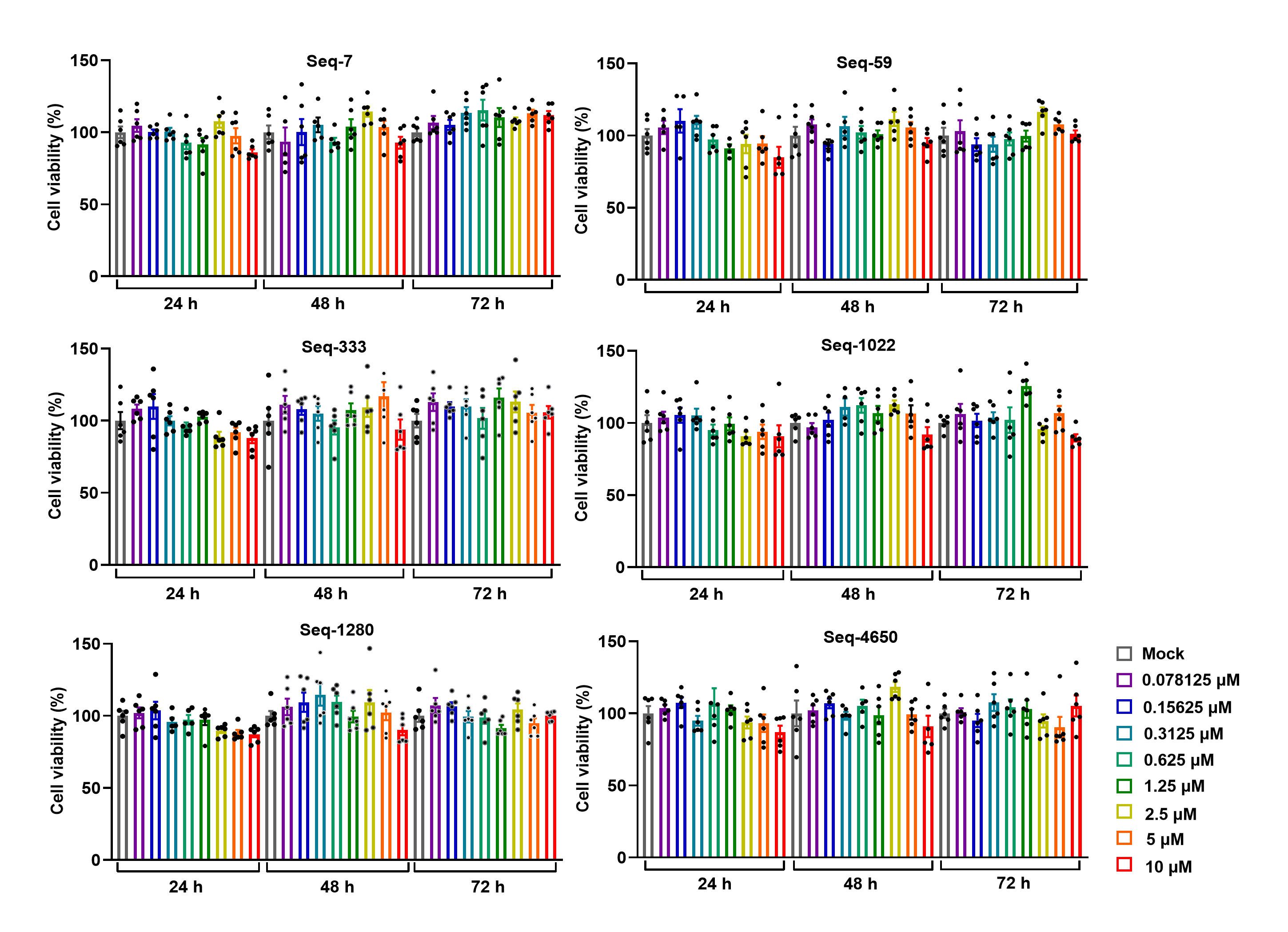


**Supplementary Figure 8.** **Cytotoxicity of six aptamers evaluated by CCK-8 at different time point in 16HBE cells.** n=3 per group, all data are shown as the mean ± SEM.


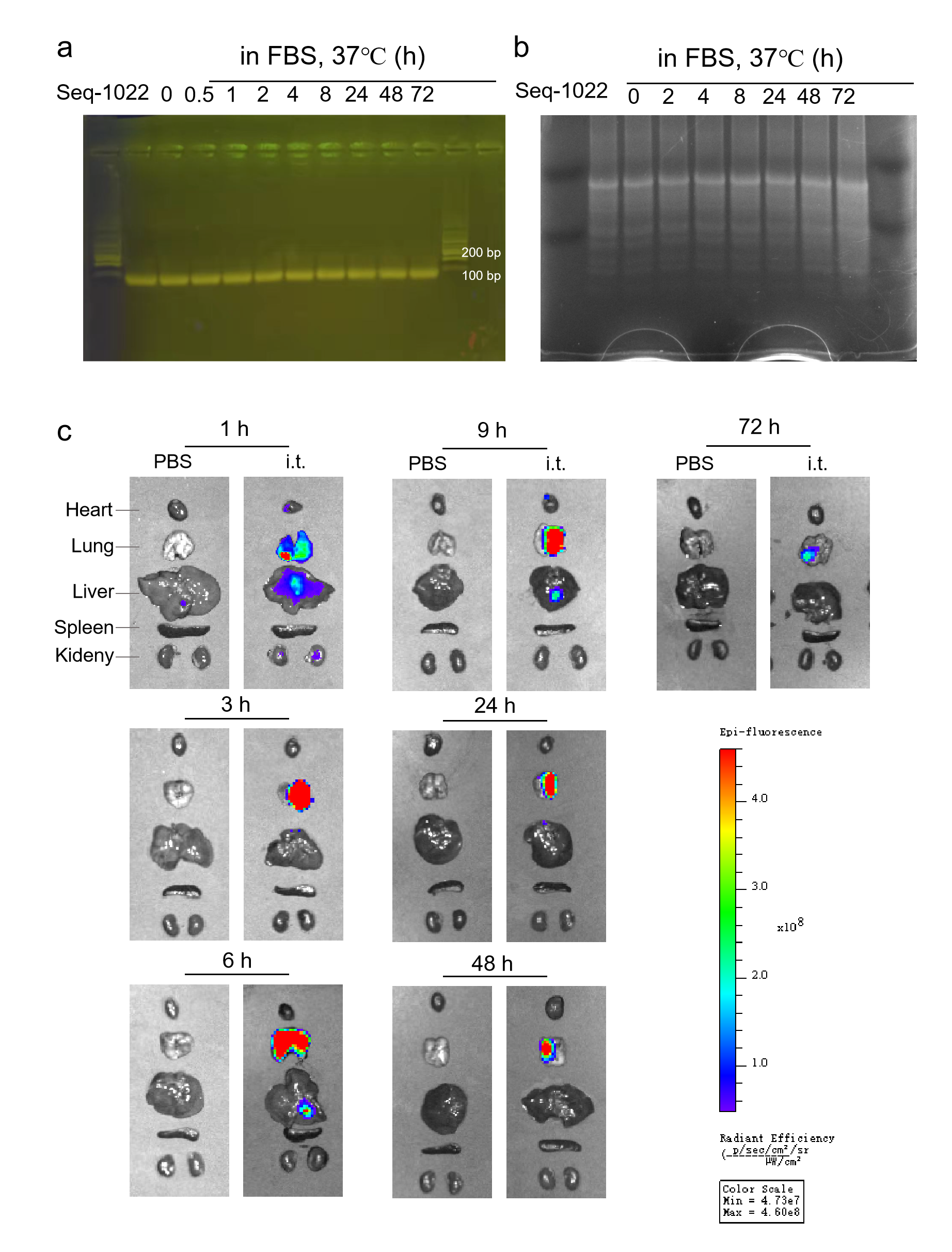


**Supplementary Figure 9.** **Stability analysis of Seq-1022** in (a) agarose gel and (b) PAGE, Aptamer Seq-1022 incubated with cell media (containing 10% FBS) at 37°C for different times. (c) Fluorescence distribution in mice of Cy5-labeled Seq-1022 by intratracheal injection for different times.


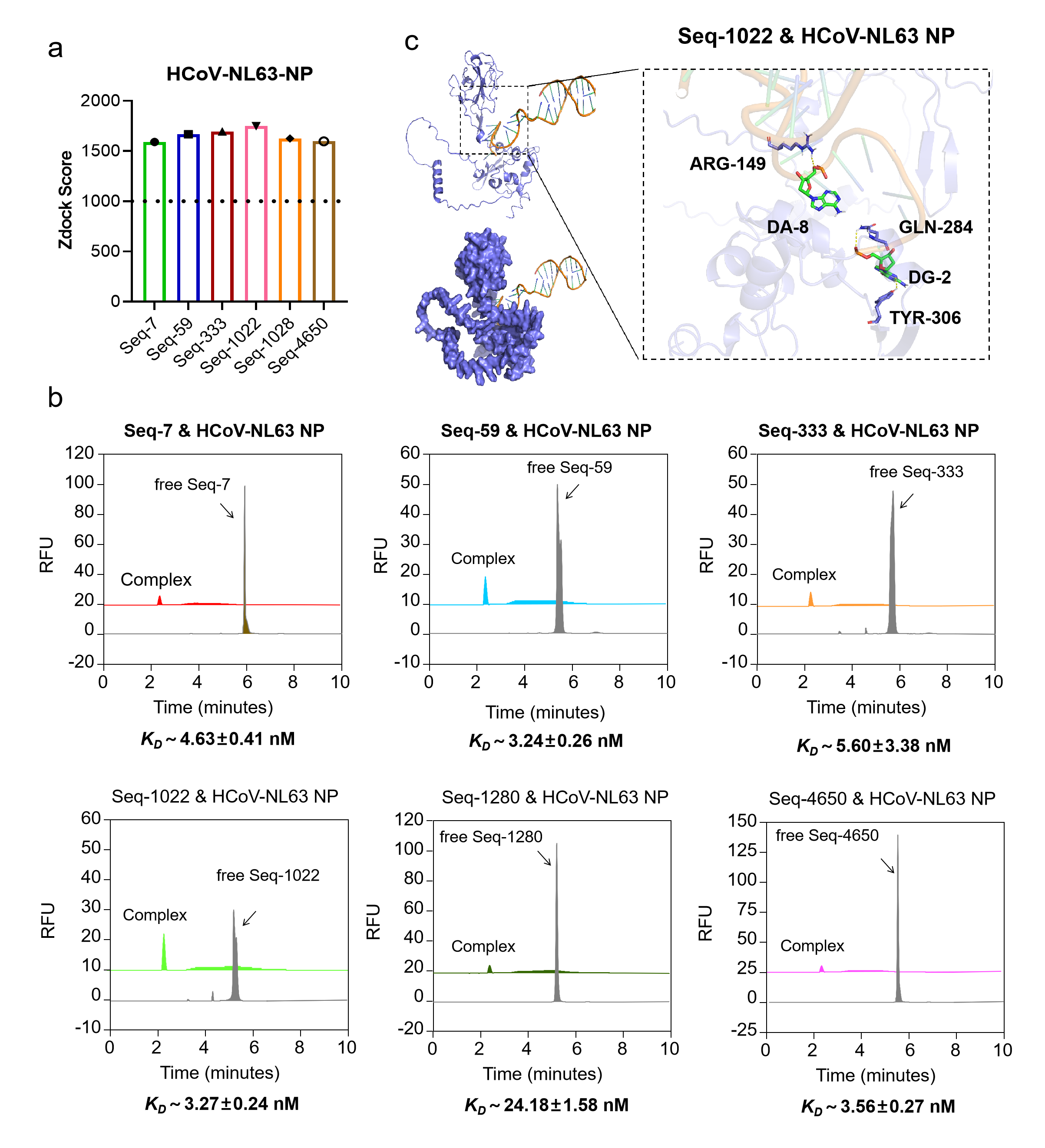


**Supplementary Figure 10.** **Prediction of binding and affinity identification between the aptamers and the N protein of -NL63.** **(a)** Prediction of the binding affinity between the aptamers and NP of -NL63 via Zdock score. **(b)** The characterizations of affinity and specificity between aptamers and NP of -NL63 were performed via CE. **(c)** The results of molecular docking to predict and simulate the binding sites of aptamer Seq-1022 to NP of -NL63 using Discovery Studio software.


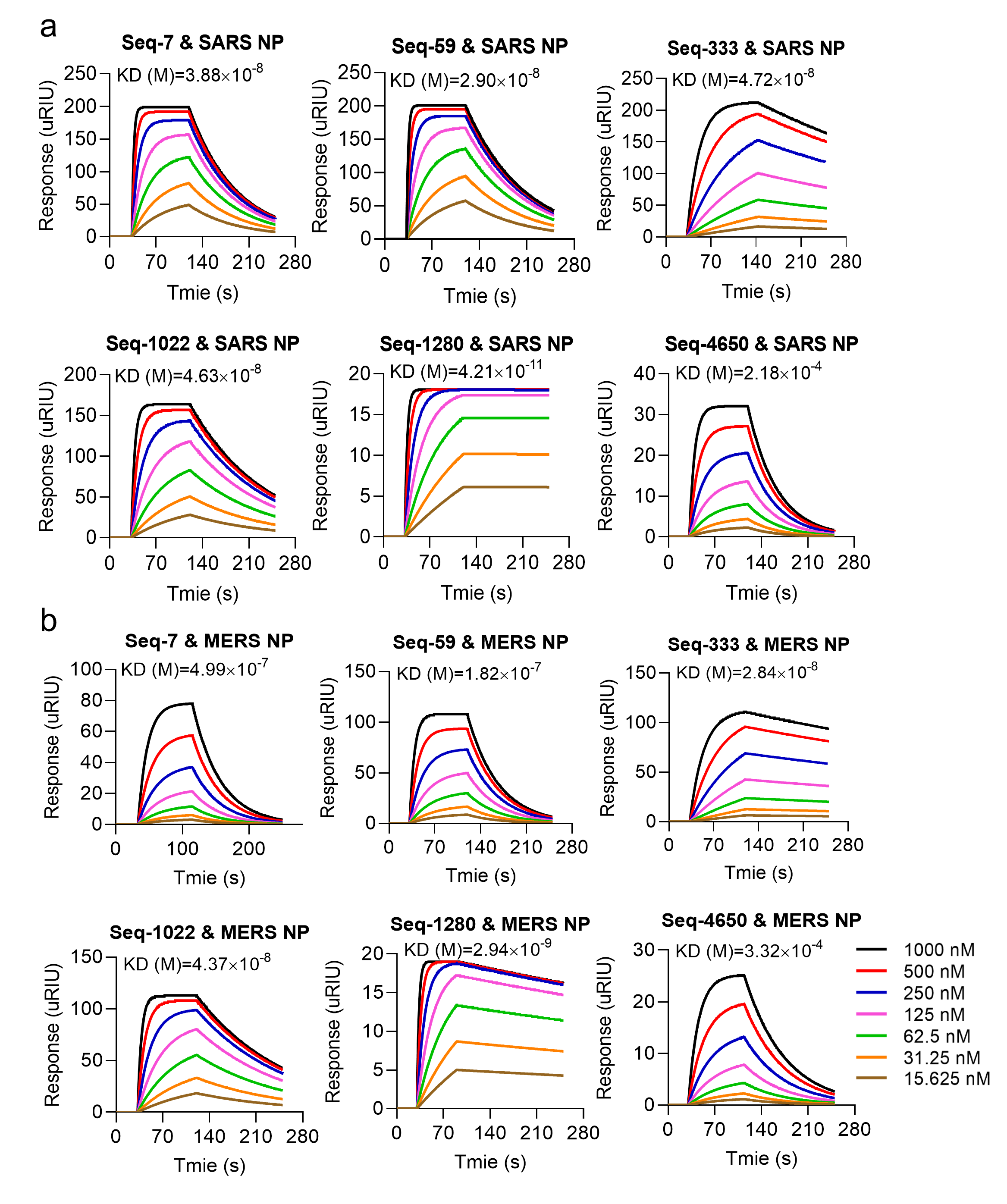


**Supplementary Figure 11. Affinity identification between the aptamers and the N proteins** of **(a)** SARS and **(b)** MERS by SPR.


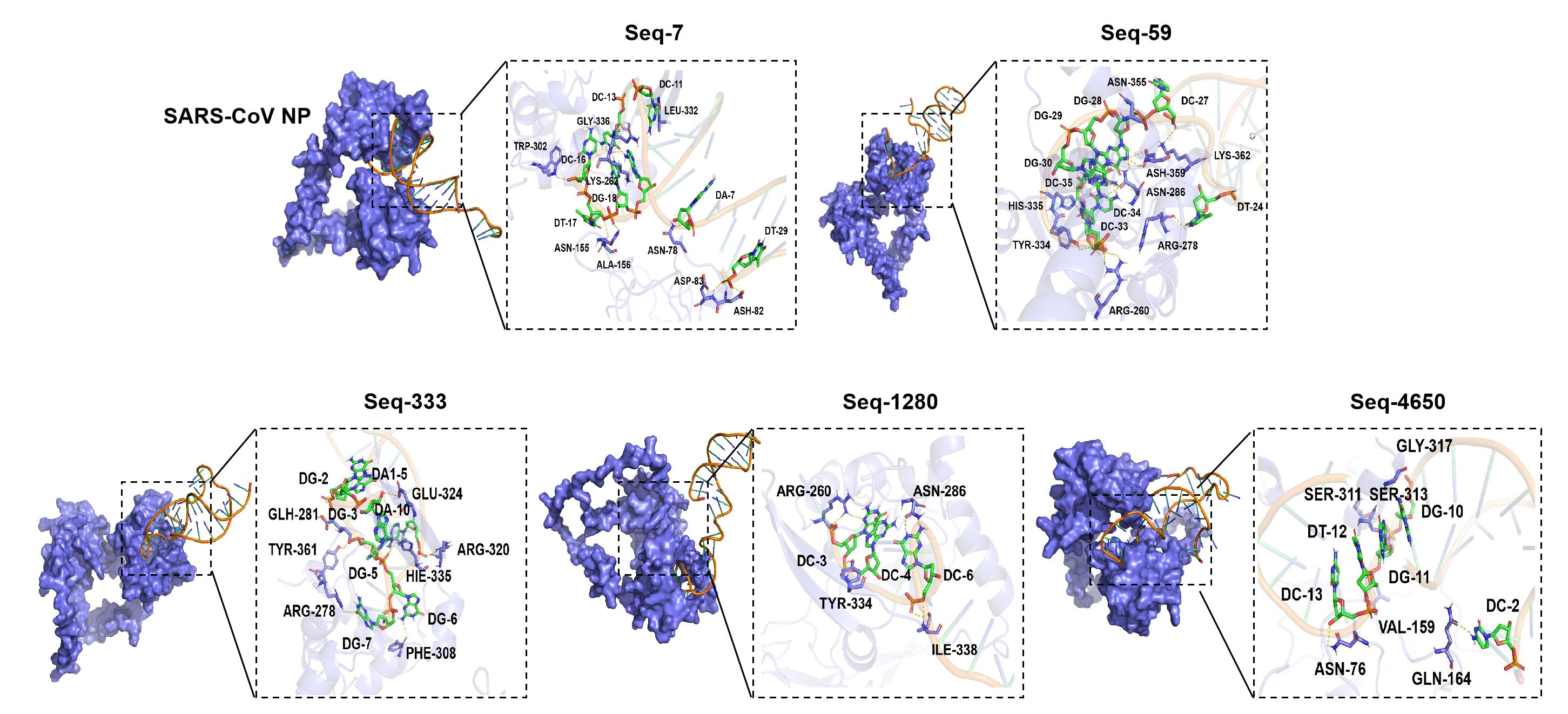


**Supplementary Figure 12. Binding sites of aptamers with SARS NP predicted by molecular docking.**


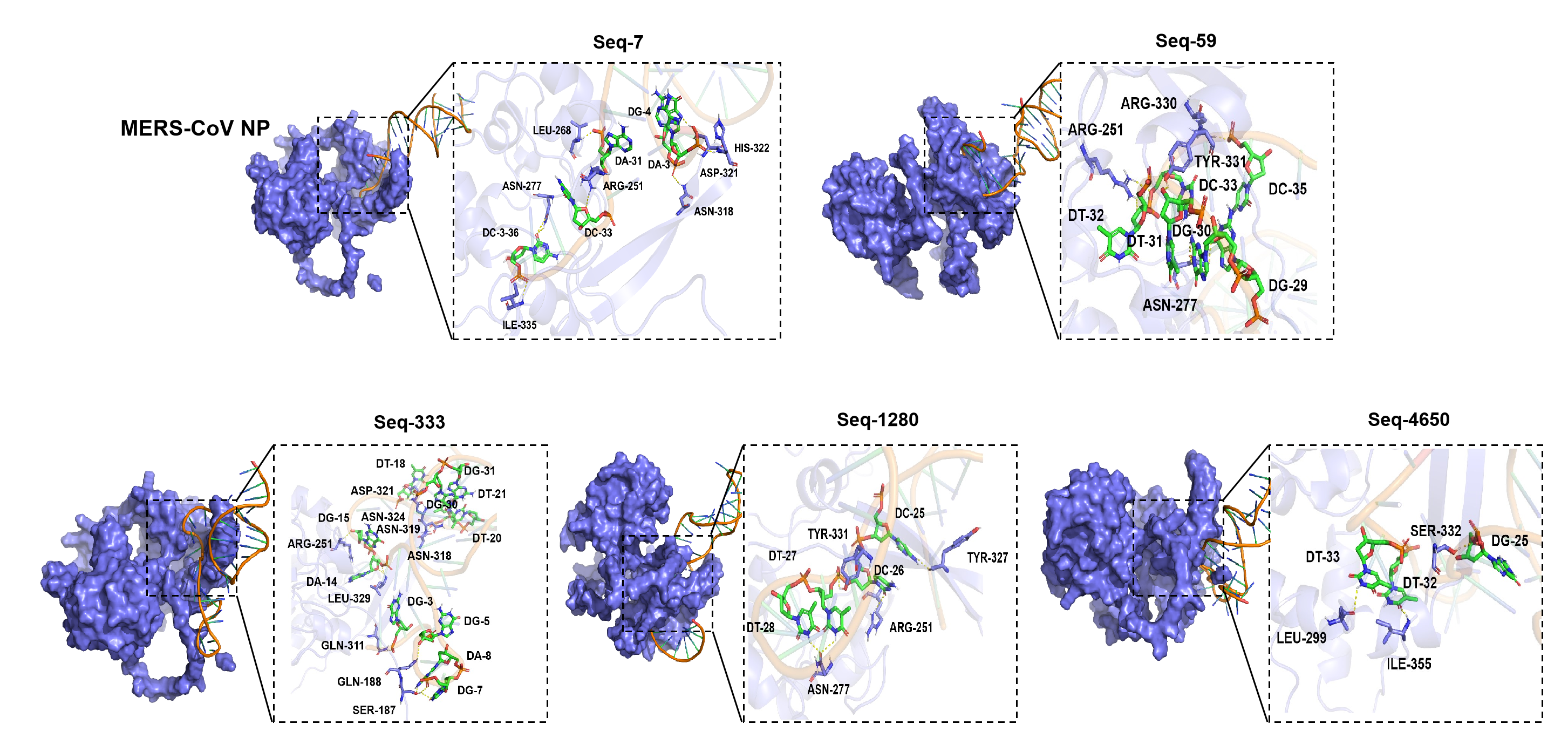


**Supplementary Figure 13. Binding sites of aptamers with MERS NP predicted by molecular docking.**


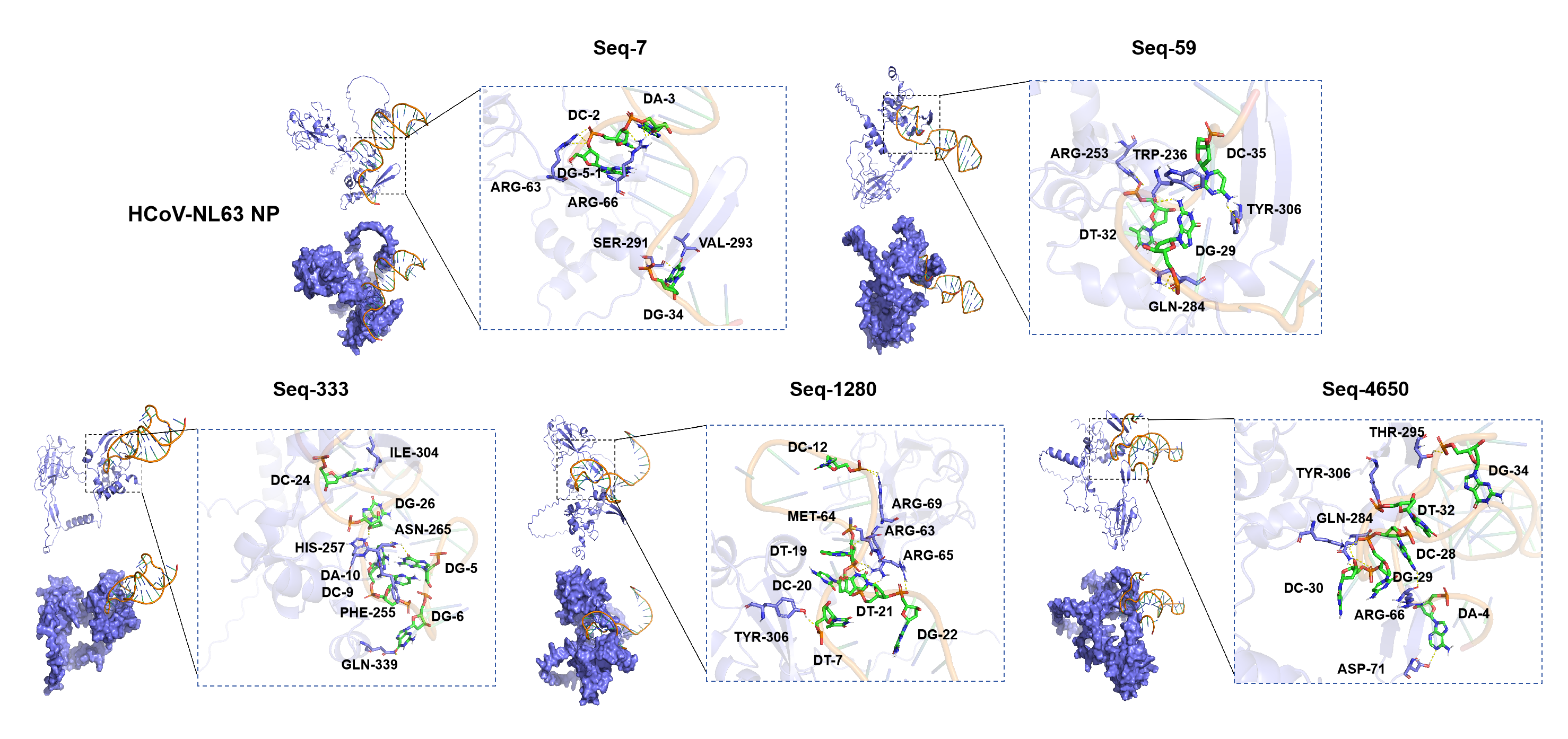


**Supplementary Figure 14. Binding sites of aptamers with HCoV-NL63 NP predicted by molecular docking.**

**
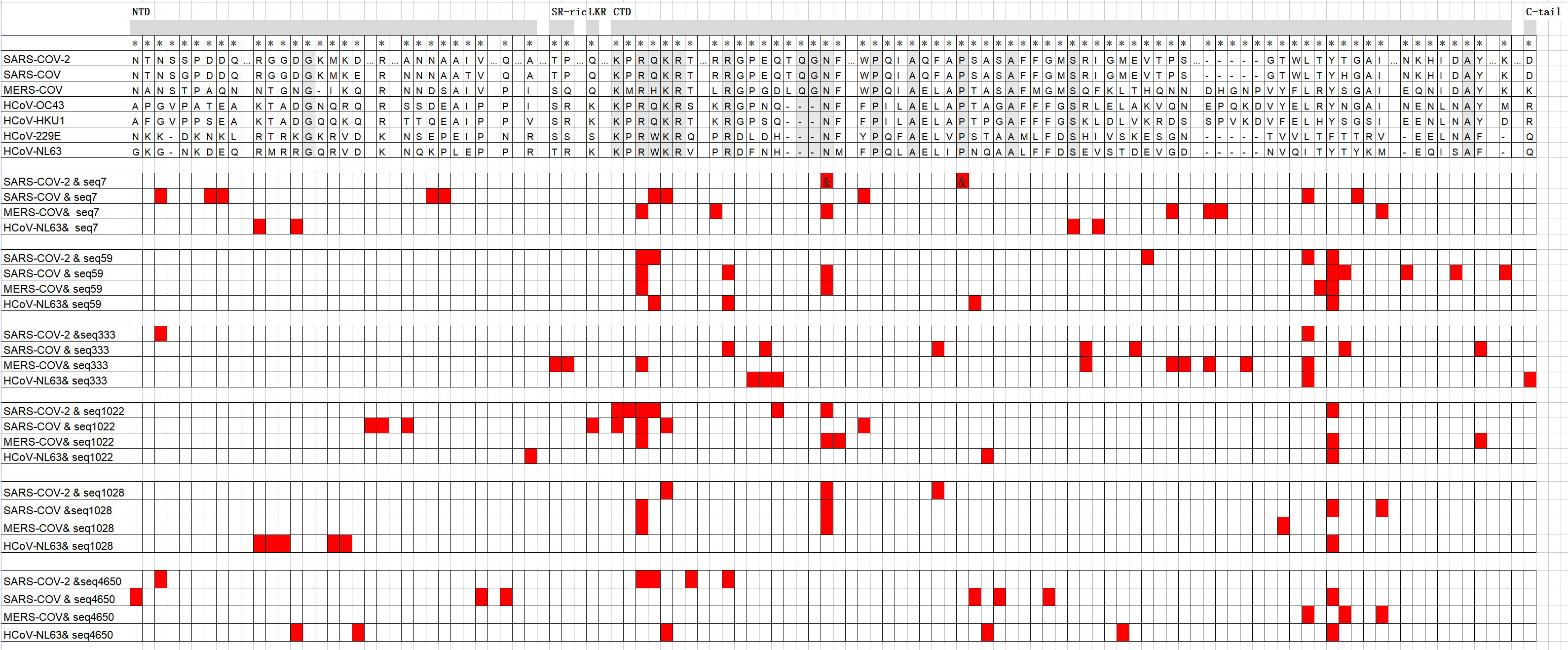
Supplementary Figure 15. Prediction and conservation analysis of binding sites of aptamers with seven HCoVs.**

**
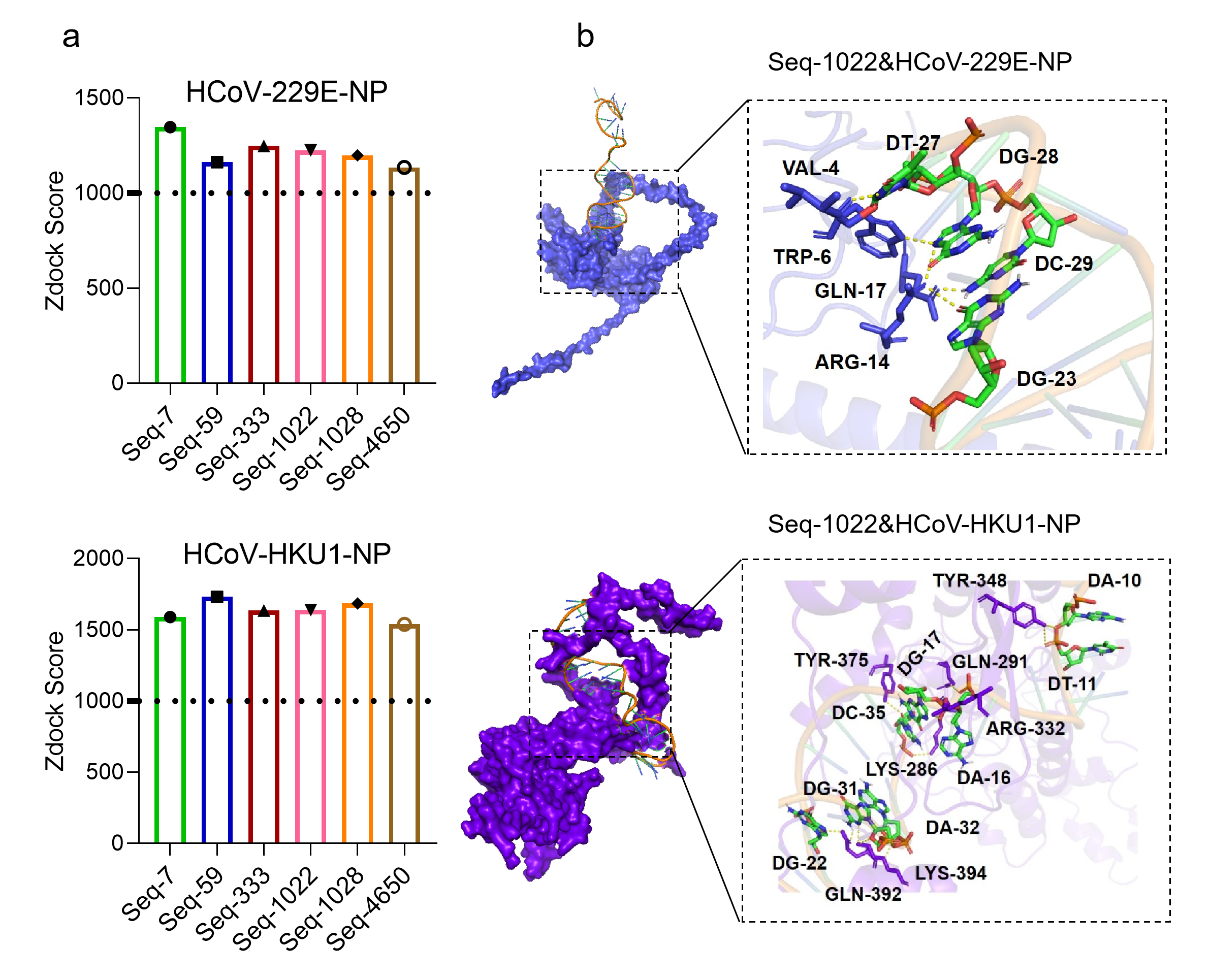
**

**Supplementary Figure 16. Prediction of the binding affinity between the aptamers and other HCoVs N proteins** using Zdock score **(a)** and the binding sites of Seq-1022 with NP predicted by molecular docking **(b)**.

**Supplementary Tables**

**Supplementary Table 1. Aptamers sequences and its screening information.**

| Aptamer | Sequence (5'-3') | Screening  time | Screening  frequency | Cycle Threshold Value |
| --- | --- | --- | --- | --- |
| Seq-335 | AGGGGGGACATCTGGATTGATGGCCGTGCGGATACG | 274 | 0.0001 | 15.709 |
| Seq-915 | TCGTGCGGATACGGTGTGGCTGTTCAGTCACAGGGG | 89 | 0 | 15.78 |
| Seq-59 | ACTCCCACCTTTATTGAGGGCGGTGACGGGTTCCCTC | 1555 | 0.0005 | 17.969 |
| Seq-296 | GGGGGAGGGGTTGGGTTGCCAGGGGCCCAGTAGTTG | 304 | 0.0001 | 16.993 |
| Seq-771 | AGGGGGGACATCTGGATAGTTGGCCGTGCGGATACG | 108 | 0 | 15.326 |
| Seq-391 | CCACCTGCTGCTGCATCTCACGGTCTGGTCCCCTCCG | 230 | 0.0001 | 15.359 |
| Seq-769 | GCAGGCAAGGGGTTGGGTTCCTGCTAAGGCTTCGTTG | 108 | 0 | 16.003 |
| Seq-89 | AGGGGGGACATCTGGATTGGTGGCCGTGCGGATACG | 987 | 0.0003 | 15.169 |
| Seq-409 | GCAGGCAAGGCTCTACCGACCCGTTGCTTGATCGAC | 222 | 0.0001 | 15.186 |
| Seq-461 | ACAGGCAAGGCTCTACTGACCCGTTGCTTGATCGAC | 194 | 0.0001 | 16.201 |
| Seq-930 | GCCATAACCACGGTTGCTGGCTCCTTCCCTCCTACTC | 87 | 0 | 16.237 |
| Seq-879 | CGGCTTGCCCGTCCAGAACCTGTGTTCGTTCATCTT | 93 | 0 | 17.168 |
| Seq-277 | CACGCACGGTCTGGGCCCCGTCATCGCTTCATGCTCC | 320 | 0.0001 | 17.006 |
| Seq-7 | GCAGGCAAGGCTCTACTGACCCGTTGCTTGATCGAC | 47757 | 0.0156 | 18.818 |
| Seq-19 | GCAGGCAAGGCTCTACTGACCCGTTGCTTGATCAAC | 10571 | 0.0035 | 16.937 |
| Seq-239 | GCAGGCAAGGCTCTACTGTCCCGTTGCTTGATCGAC | 367 | 0.0001 | 15.004 |
| Seq-278 | GCAGGCAAGGCTCTACTGACTCGTTGCTTGATCGAC | 320 | 0.0001 | 14.787 |
| Seq-734 | GCAGGCAAGGCTCTACTGTCCCGTTGCTTGATCAAC | 113 | 0 | 17.549 |
| Seq-823 | GCAGGCAAGGCTCTATTGACCCGTTGCTTGATCGAC | 99 | 0 | 15.004 |
| Seq-997 | GCAGGCAAGGCTCTACTGACCCGTTGCTTGATCGGC | 79 | 0 | 15.669 |
| Seq-574 | CCCCTTGGCTCTCCACGGTAACTTGCTGTTGTCCCCTT | 152 | 0 | 15.736 |
| Seq-333 | AGGGGGGACACCTAGATTGTTGGCCGTGCGGATACG | 274 | 0.0001 | 17.969 |
| Seq-359 | AGGGGGGACATCTGGATTGCTGGCCGTGCGGATACG | 253 | 0.0001 | 17.125 |
| Seq-419 | AGGGGGGACATCTAGATTGCTGGCCGTGCGGATACG | 214 | 0.0001 | 16.802 |
| Seq-608 | AGGGGGGACACCTGAATTGTTGGCCGTGCGGATACG | 141 | 0 | 17.145 |
| Seq-17 | CGCACATGGCTTTAGAGGTTCCATAGGGTACACTCT | 12353 | 0.004 | 14.904 |
| Seq-881 | GCCTGTTAGATTGGCGCGGGGTAAGGGGTTGGGTTCCTGCTAAGGCTTCGTTG | 93 | 0 | 16.456 |
| Seq-316 | ACCACGGTCCTTCAGCTGGTCCACTGGTCCTTCGCC | 290 | 0.0001 | 16.206 |
| Seq-83 | GGGGAGGGGGTTGGGTTGCCAGGGGCCTAGTAGTTG | 1133 | 0.0004 | 16.104 |
| Seq-489 | CCCGCACACCTCAGTCACGGTCTCTGGTCCATCCTC | 180 | 0.0001 | 14.853 |
| Seq-279 | CCCCCCACACTTTTCGCACGGCTGCTGTGTCCCCTCC | 319 | 0.0001 | 14.838 |
| Seq-84 | GCCTGTTAGATTGGCGCGGGGGGAGGGGTTGGGTTGCCAGGGGCCTAGTAGTTG | 1084 | 0.0004 | 14.714 |
| Seq-519 | GCCTGTTAGATTGGCGCGGGGGAAGGGGTTGGGTTGCCAGGGGCCTAGTAGTTG | 169 | 0.0001 | 14.68 |
| Seq-379 | GCCTGTTAGATTGGCGCGGGGGAAGGGGTTGGGTTCCTGCTAAGGCTTCGTTG | 237 | 0.0001 | 14.751 |
| Seq-1022 | AGGGGGGACATCTGGAGTGTTGGCCGTGCGGATACG | 77 | 0 | 18.748 |
| Seq-1280 | CACCTCTCACCCCACGGTTCTGGTCCTTCCCCGACG | 60 | 0 | 17.864 |
| Seq-2674 | GCCACGGTGGTTCTGCTGGGACCTTTGCTGGTCCTTT | 27 | 0 | 17.145 |
| Seq-4650 | TCCACGGTCGGTCCCCTTACCACTGGTCCCTTTGTC | 14 | 0 | 17.604 |

**Supplementary Table 2. Primers used in this study.**

| Primer | Sequence (5’-3’) |
| --- | --- |
| Lib1s1 | GGGACCAGCACACGCATAAC |
| Lib1s2 | CACGGTAGCACGCATAACAC |
| Symmetric PCR-F | CAGGGGACGCACCAAGG |
| Symmetric PCR-R | ATCACGCAGCACGCGGGTCATGG |
| Asymmetric PCR-F | CAGGGGACGCACCAAGG |
| Asymmetric PCR-R | CGATGTCAGCACGCGGGTCATGG |
